# Non-canonical two-step biosynthesis of anti-oomycete indole alkaloids in Kickxellales

**DOI:** 10.1186/s40694-023-00166-x

**Published:** 2023-09-05

**Authors:** Johannes Rassbach, Nathalie Hilsberg, Veit G. Haensch, Sebastian Dörner, Julia Gressler, Robin Sonnabend, Caroline Semm, Kerstin Voigt, Christian Hertweck, Markus Gressler

**Affiliations:** 1 Faculty of Biological Sciences, Pharmaceutical Microbiology, Friedrich Schiller University Jena, Winzerlaer Strasse 2, 07745 Jena, Germany; 2 Pharmaceutical Microbiology, Leibniz Institute for Natural Product Research and Infection Biology-Hans-Knöll-Institute, Winzerlaer Strasse 2, 07745 Jena, Germany; 3 Biomolecular Chemistry, Leibniz Institute for Natural Product Research and Infection Biology-Hans-Knöll-Institute, Adolf-Reichwein-Strasse 23, 07745 Jena, Germany; 4 Faculty of Biological Sciences, Institute of Microbiology, Friedrich Schiller University Jena, Neugasse 25, 07743 Jena, Germany; 5 Jena Microbial Resource Collection (JMRC), Leibniz Institute for Natural Product Research and Infection Biology-Hans Knöll Institute, Adolf-Reichwein-Strasse 23, 07745 Jena, Germany

**Keywords:** Early-diverging fungi, Secondary metabolite, Indole alkaloid, *Linderina pennispora*, CoA ligase, Transferase, Indole-3-acetic acid

## Abstract

**Background:**

Fungi are prolific producers of bioactive small molecules of pharmaceutical or agricultural interest. The secondary metabolism of higher fungi (Dikarya) has been well-investigated which led to > 39,000 described compounds. However, natural product researchers scarcely drew attention to early-diverging fungi (Mucoro- and Zoopagomycota) as they are considered to rarely produce secondary metabolites. Indeed, only 15 compounds have as yet been isolated from the entire phylum of the Zoopagomycota.

**Results:**

Here, we showcase eight species of the order Kickxellales (phylum Zoopagomycota) as potent producers of the indole-3-acetic acid (IAA)-derived compounds lindolins A and B. The compounds are produced both under laboratory conditions and in the natural soil habitat suggesting a specialized ecological function. Indeed, lindolin A is a selective agent against plant-pathogenic oomycetes such as *Phytophthora* sp. Lindolin biosynthesis was reconstituted in vitro and relies on the activity of two enzymes of dissimilar evolutionary origin: Whilst the IAA–CoA ligase LinA has evolved from fungal 4-coumaryl-CoA synthetases, the subsequently acting IAA-CoA:anthranilate *N*-indole-3-acetyltransferase LinB is a unique enzyme across all kingdoms of life.

**Conclusions:**

This is the first report on bioactive secondary metabolites in the subphylum Kickxellomycotina and the first evidence for a non-clustered, two-step biosynthetic route of secondary metabolites in early-diverging fungi. Thus, the generally accepted “gene cluster hypothesis” for natural products needs to be reconsidered for early diverging fungi.

**Supplementary Information:**

The online version contains supplementary material available at 10.1186/s40694-023-00166-x.

## Introduction

Early diverging fungi (EDF) are a comparatively novel resource of natural products [1], whereas higher fungi (i.e. Asco- and Basidiomycota) are a well-established reservoir for natural compounds of pharmaceutical relevance. In the post-genomic era an intensive reclassification of EDF has begun and is still an on-going process [1–5]. Following current taxonomy, filamentous EDF divide into two major phyla [6]. The Mucoromycota comprise well-characterized genera such as *Mucor*,* Rhizopus*,* Phycomyces* and *Mortierella* encompassing plant symbionts and potent producers of industrially relevant polyunsaturated fatty acids and β-carotene-derived pigments [7, 8]. Some of these fungi produce nonribosomal peptides (NRP) with surface-active, antibacterial or antimalarial activities [9–13]. In contrast, the second phylum, the Zoopagomycota, is more nuanced as they include (i) obligatory pathogens of invertebrates or amoebae (Zoopagomycotina), along with (ii) parasites and commensals of insects or amphibians (Entomophthoromycotina), and (iii) mycoparasites or saprotrophic species (Kickxellomycotina) [2, 6, 14].

Saprotrophic kickxellomycetes are rarely isolated from nature, but preferably from rhizosphere, soil, humus, dung, and other organic material from dead plants and animals [2, 15]. Colonies usually grow slowly which is why they are quickly covered by other saprobe ascomycetes prior to isolation [15]. With at least 167 isolated and ITS sequenced species, *Coemansia* is the major genus among the subphylum Kickxellomycotina [2]. In contrast, the rarely isolated kickxellomycetes include *Linderina* species (with *L. pennispora* and *L. macrosporum* as the sole identified species) [16], *Martensiomyces pterosporus* [17] and *Kickxella alabastrina* [18, 19]. However, although they are easily cultivable representatives of the Zoopagomycota, neither their metabolic potential nor their ecological impact have been investigated yet.

We addressed this profound knowledge gap. Here, we report that *L. pennispora, M. pterosporus,* and six *Coemansia* species are producers of bioactive indole alkaloids. The indole-3-acetic acid (IAA)-derived anthranilic amides, lindolin A and B, are secreted to the culture supernatant and during growth in soil organic matter suggesting an ecological relevance. Indeed, lindolins possess moderate, but selective activity against the plant-pathogenic oomycete *Phytophthora megasperma* whilst other antimicrobial, phyto- or cytotoxic side activities were not detected. The combined activities of the IAA–CoA ligase LinA and the unique IAA-CoA:anthranilate *N*-indole-3-acetyltransferase LinB catalyze in the NRP synthetase (NRPS)-independent amide formation of lindolins. The transferase LinB is a unique enzyme and highly conserved among all Kickxellales indicating lindolin biosynthesis as a chemotaxonomic marker of this prolific fungal order.

## Results

### The secondary metabolism of the fungal division Zoopagomycota is underexplored

**Fig. 1:**
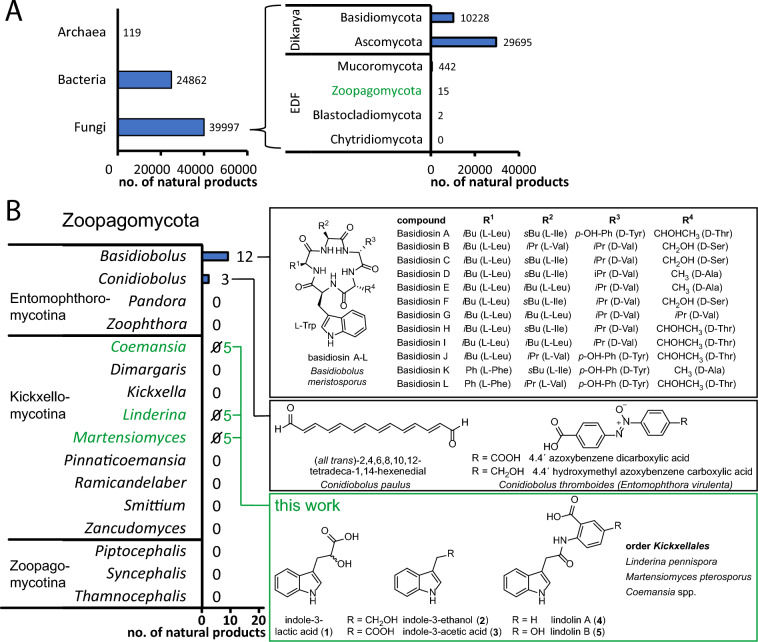
Overview of known metabolites of early diverging fungi (EDF) and Zoopagomycota. **A** The metabolite count was extracted from all microbial kingdoms and fungal orders from the LOTUS database and was additionally manually inspected for EDF [20]. **B** Metabolites from the fungal phylum Zoopagomycota are rare and include peptides [21–23], polyenes [24] and azoxyaromates [25]. Note, that this survey is non-exhaustive as kingdom-wide common primary metabolites such as unmodified sesquiterpenes, fatty acids and their degradation products are not included

Initially, we screened the LOTUS natural product data bank for secondary metabolite (SM) producers among the EDF [20]. As expected, higher fungi (Dikarya) have been intensively studied and dominated the database with approx. 40,000 fungal compounds by far (Fig. 1A). However, EDF are hardly known to produce SMs as merely 1% of the fungal metabolites (459 compounds) are EDF-derived. The most prominent producers are species from the Mucoromycota (442 compounds), whilst in sum solely 17 metabolites have been isolated from the three major phyla Zoopagomycota*,* Blastocladiomycota and Chytridiomycota altogether (Fig. 1B).

This contrasts the number of biosynthetic genes for polyketides, nonribosomal peptide synthetases (NRPS) and terpene cyclases that were identified in EDF throughout the 1000 fungal genomes project and other studies [1, 26–28]. As Zoopagomycota are paraphyletic, unique biosynthetic enzymes and hence novel metabolites with unusual activities are expected [29]. Therefore, re-discovery of known compounds—as frequently observed in the evolutionarily distantly related Dikarya species—is unlikely [30]. Only 15 natural products, i.e. 12 cyclopentapeptides from *Basidiobolus meristosporus* [21–23], two azoxybenzene derivatives from *Conidiobolus thromboides *(*syn. Entomophthora virulenta*) [25] and the yellow pigment (*all trans*)-2,4,6,8,10,12-tetradeca-1,14-hexenedial from *Conidiobolus paulus* [24] constitute the entire set of currently known Zoopagomycota compounds (Fig. 1B). Hence, Zoopagomycota are a promising, but underinvestigated source of novel natural products.

### Production of indole-3-acetamides is specific for the order Kickxellales

**Fig. 2:**
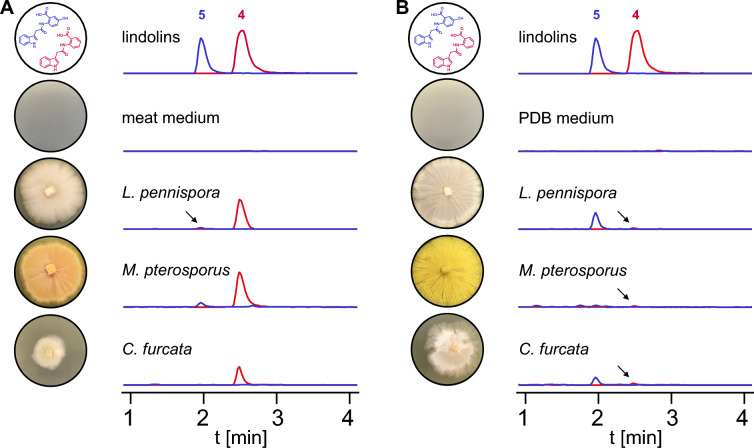
Production of lindolin A and B in Kickxellales. Pictures of agar plates and extracted ion chromatograms (EIC) of metabolite crude extracts of *Linderina pennispora*, *Martensiomyces pterosporus* and *Coemansia furcata* grown in liquid meat medium (meat, **A**) or potato dextrose broth (PDB,** B**) at 160 rpm for 6 d. EICs were recorded at *m/z* 293 [*M*−H]^−^ and *m/z* 309 [*M*−H]^−^ for **4** (red trace) and **5** (blue trace), respectively. Non-inoculated medium served as negative controls. Arrows indicate traces of** 4** or **5**

First, we studied the secondary metabolome of *L. pennispora*, a morphologically well-described, saprotrophic species of the order Kickxellales (Additional file 1: Table S1) [31, 32]. We detected five UV-active compounds (**1**–**5**) (Fig. 1B), of which **4** and **5** with corresponding ion masses of *m/z* 293.0927 [*M*−H]^−^ (calc. *m/z* 293.0931 [*M*−H]^−^ for C_17_H_13_N_2_O_3_^−^) and *m/z* 309.0881 [*M*−H]^−^ (calc. *m/z* 309.0880 [*M*−H]^−^ for C_17_H_13_N_2_O_4_^−^), respectively, dominated in the culture filtrate extracts (Fig. 2A). Compound isolation by flash chromatography and HPLC as well as subsequent MS/MS analysis and NMR spectroscopy-based structure elucidation revealed a new natural product, lindolin A (**4**), as an amide of indole-3-acetic acid (IAA) and anthranilic acid (Additional files 2, 3, 4, 5, 6, 7, 8, 9, 10, 11: Figure S1–S9, Table S2). Lindolin B (**5**) was assigned as a derivative of **4** with a 5ʹ-hydroxy group at the anthranilic moiety (Additional files 12, 13, 14, 15, 16, 17, 18, 19, 20, 21: Figure S10–S18, Table S3). Additionally, we detected indole-3-acetic acid (IAA, **3**) and minor amounts of the **3**-related compounds indole lactic acid (ILA, **1**) and indole ethanol (IOL, **2**) in the extracts as determined by GC–MS/MS using a trimethylsilyl derivatization procedure of commercially available or synthesized reference standards (Fig. 1B, Additional file 22: Figure S19, Additional file 23: Figure S20, Additional file 24: Figure S21). Next, we screened the secondary metabolomes of more distantly related Kickxellales [2] such as *Martensiomyces pterosporus,* and six *Coemansia* species. Interestingly, all tested species synthesized **4** and **5**, at which *M. pterosporus* and *Coemansia furcata* (syn*. formosensis*) [33] were the most prominent lindolin producers (Fig. 2 and Additional file 25: Figure S22). Although **4** and **5** were detectable under any cultivation condition, the 5ʹ-hydroxy derivative **5** was mainly produced on glucose-rich media (Fig. 2B). Additionally, we exemplarily detected **1** and** 3** in *M. pterosporus* and **1–3** in *C. furcata* by GC–MS (Additional file 22: Figure S19, Additional file 23: Figure S20, Additional file 24: Figure S21). This suggests, that (i) **4** and** 5** are derived from **3** in all tested species and (ii) **1** and **2** are byproducts of the fungal **3** biosynthesis pathway. In contrast to the Kickxellales, species of the related kickxellomycete order Dimargaritales (*Dimargaris bacillispora*) neither produce **3** nor lindolins (**4**–**5**) (Additional file 25: Figure S22). Hence, the biosynthesis of lindolins is highly specific to the Kickxellales, but widely distributed within them. Since lindolins have never been described from other species, the compounds can be considered as specific chemotaxonomic markers for this particular fungal order.

### Lindolins are produced in soil

**Fig. 3:**
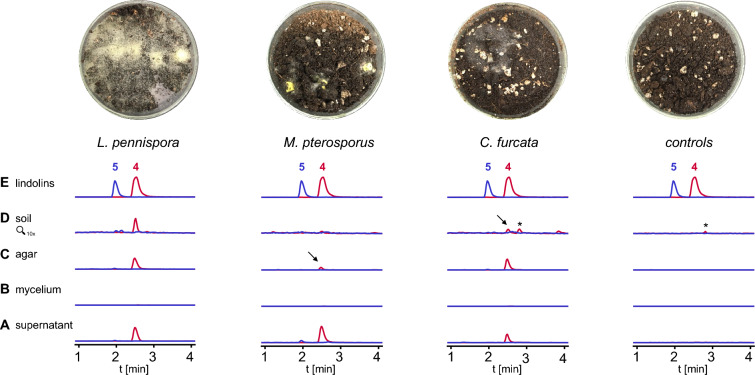
Secretion of lindolins under natural conditions. *Linderina pennispora*, *Martensiomyces pterosporus* and *Coemansia furcata* were submersely grown in liquid meat medium under agitation. Crude extracts of culture supernatants (**A**) and mycelia (**B**) were chromatographed. In addition, cultures were emersely cultivated on meat agar plates (**C**) and in potting soil supplemented with d-glucose (**D**). Authentic standards of **4** and **5** served as controls (**E**). Pictures of the soil cultures are shown above the chromatograms (Aerial mycelium is mainly visible for *L. pennispora* and *C. furcata*. Minor white spots in the control plates are part of the soil substrate). Chromatograms were recorded by UHPLC-MS and overlaid extracted ion chromatograms were shown for *m/z* 293 [*M*−H]^−^ and *m/z* 309 [*M*−H]^−^ for **4** (red trace) and **5** (blue trace), respectively. Non-inoculated media or soil served as negative controls. Asterisks indicate an unrelated compound that is also present in the soil negative control. Note, that lindolin A (**4**) is absent in the mycelium, but is the predominant secreted compound in the supernatant, agar plates and soil

Lindolins are preferably isolated from the supernatant of submerse cultures in *L. pennispora*, *M. pterosporus* and *C. furcata*, but are not detectable in mycelia, suggesting that the compounds are actively secreted (Fig. 3A, B) Similarly, emerse cultures (agar plates) led to the production of lindolins (Fig. 3C). However, cultivation in flasks and on plates are highly artificial growth conditions and do not resemble the natural habitat. Though, *L. pennispora*, *M. pterosporus* and *C. furcata* were additionally cultivated in potting soil in presence of d-glucose to boost the fungal growth (Fig. 3D). Again, **4** and **5** were detectable in at least two of the three species indicating that lindolins are produced in nature by Kickxellales and may have an impact in modulating their ecological niche.

### Lindolins are anti-oomycete compounds

We screened for a potential ecological function of lindolins with various biological assays. Lindolins are neither antibacterial compounds nor cytotoxic against mammalian cells (Additional file 26: Figure S23 and Additional file 27: Figure S24). As kickxellomycetes were isolated from grasslands [34] and fungal growth is positively correlated with a high carbon-to-nitrogen ratio [35], we studied the impact of lindolins on plant growth: Because lindolins share structural similarities to the major plant hormone auxin (indole-3-acetic acid, IAA, **3**) [36], we specifically determined root growth inhibitory activity of the compounds using radish seedlings (Additional file 28: Figure S25). However, lindolins (**4**–**5**) neither induce root shortening as **3** nor impairs **3**-mediated plant growth in general. Hence, at least on the tested seedlings, lindolins do not show phytotoxic activities. However, during our initial screening we recognized an activity of lindolins against the plant pathogenic oomycete *Phytophthora megasperma*, while plant pathogenic fungi (e.g. *Fusarium graminearum*) were unaffected (Additional file 29: Figure S26). **4** showed moderate activity against oomycetes *P. megasperma*, (MIC_50_ of 100 ± 11 µM) and—to a lower extend—*Pythium macrosporum* (MIC_50_ 215 ± 61 µM), whilst **5** did not inhibit oomycete growth (MIC_50_ > 500 µM), suggesting that the 5ʹ-hydroxylation dramatically impairs bioactivity (Additional file 30: Figure S27).

### In silico reconstruction of the lindolin biosynthetic pathway

**Fig. 4:**
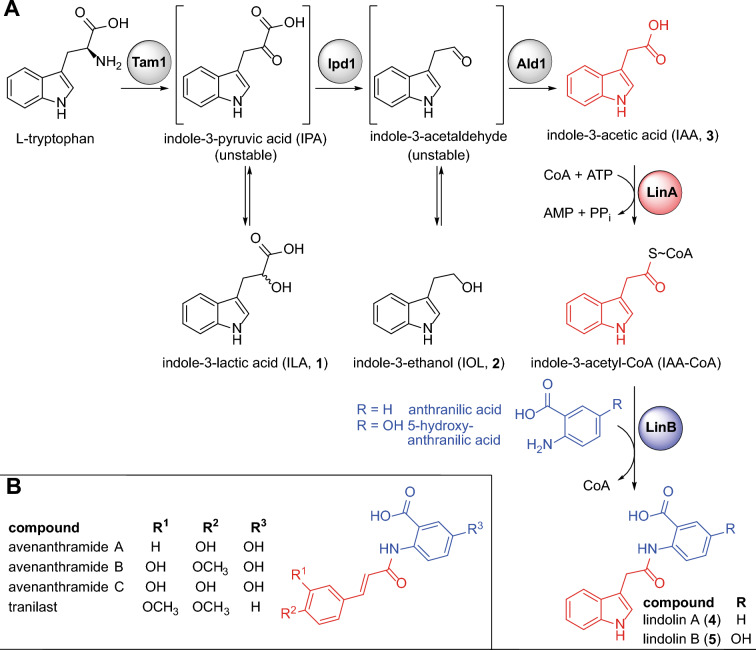
Lindolin biosynthesis in *L. pennispora* and other Kickxellales. **A** IAA and lindolin biosyntheses build on l-tryptophan via two unstable intermediates (brackets). Predicted enzymes are shown as grey spheres. Biochemically verified enzymes, i.e. the indole-3-acetic acid-CoA ligase LinA and the indole-3-acetyl-CoA:anthranilate *N*-indole-3-acetyltransferase LinB, are shown in red and blue spheres, respectively. **B** Structurally related compounds from oat (avenanthramides A–C) and the antiallergic drug tranilast

In EDF, peptide compounds are usually formed by NRPSs as demonstrated for hexapeptides [9, 37] and cyclopentapeptides [38] from *M. alpina* and postulated for the cyclopentapeptides from *B. meristosporus* [21]. Commonly, small fungal amides such as fumarylalanine from the pathogenic ascomycete *Aspergillus fumigatus* [39] or benzodiazephinedione from *Neosartorya fischeri* [40] are synthesized by condensation of two (amino) acids using a bimodular NRPS. However, the genomes of *L. pennispora* and related Kickxellales species lack genes for bimodular NRPSs [2]. Therefore, we postulated a coenzyme A (CoA)-mediated route for the production of lindolins (Fig. 4A).

The precursor IAA in *L. pennispora* might be produced by a common indole-3-pyruvic acid (IPA) pathway reported for basidiomycetes [41] starting from l-tryptophan using three consecutive enzymes namely the l-tryptophan aminotransferase Tam1, the IPA decarboxylase Ipd1, and the indole-3-acetaldehyde dehydrogenase Ald1. This hypothesis is supported by three observations: (i) the genomes of *L. pennispora* and related Kickxellales encode similar enzymes (Additional file 31: Table S4), (ii) supplementation of the cultures with l-tryptophan boosts the production of **3** (and lindolins) 19-fold (threefold) (Additional file 32: Figure S28), and (iii) the shunt products **1** and **2** of the highly unstable α-keto- and aldehyde intermediates are detectable in the culture broth of *L. pennispora* and other Kickxellales (Additional file 22: Figure S19, Additional file 23: Figure S20, Additional file 24: Figure S21). The production of **3** (and partially **1** and **2**) has been already demonstrated for numerous basidiomycetes [41–43], ascomycetes [44, 45], but also for some EDF such as *Mortierella antarctica* [46], *Podila verticillata* [46] and *Mucor *sp*.* [47]. However, this is the first report on **3** production in Kickxellales.

**Fig. 5:**
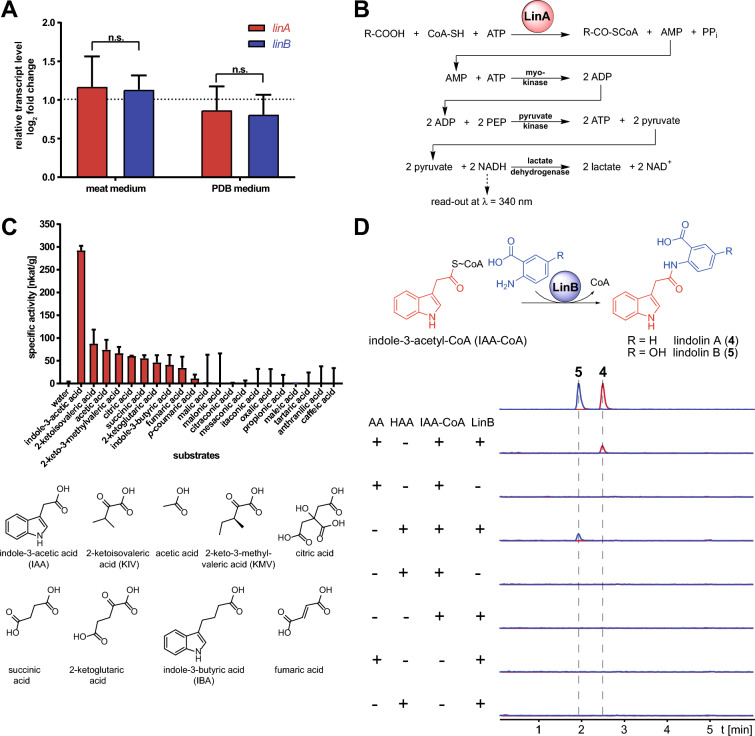
Expressional profiling of *lin* genes and enzyme activities of the ligase LinA and the transferase LinB*.* **A** Expressional profiling of *linA* and *linB* in *L. pennispora* grown in meat and PDB medium*.* The expression was referenced to growth in *Aspergillus* minimal medium and normalized against the housekeeping gene encoding the glyceraldehyde-3-phosphate dehydrogenase (*gpdA*). Both genes were co-expressed. **B** Coupled multi-enzyme assay to determine the substrate specificity of LinA. **C** Substrate profiling of LinA. All substrates were used at a final concentration of 1 mM. Water served as negative control. The assay was performed as described [49]. **D** LinB activity assay. LinB was incubated with IAA-CoA and either AA to produce **4** or HAA to produce **5**. Overlaid extracted ion chromatograms (EIC) were shown for **4** (*m/z* 293 [*M*−H]^−^, in red) and **5** (*m/z* 309 [*M*−H]^−^), in blue), respectively. Assays without enzyme or without substrates were included as negative controls. Authentic standards of **4** and **5** served as positive controls

The subsequent amidation of **3** with anthranilic acid (AA) requires two separate steps: (i) an activation of the chemically unreactive carboxylic acid of **3** by adenylation and subsequent thioesterification by a ligase and (ii) the final amidation of IAA with AA by a transferase (Fig. 4A). A similar mechanism has been shown for the avenanthramide biosynthesis in oat (*Avena sativa*) [48]. Hereby, the 4-coumarate–CoA ligase 4CL activates 4-coumaric acid to 4-coumaryl-CoA, which in turn is amidated with 5-hydroxyanthranilic acid by the hydroxycinnamoyl﻿-CoA:5-hydroxyanthranilate *N*-hydroxycinnamoyl transferase HHT1 to yield the final product avenanthramide A (Fig. 4B). We searched the genome for probable candidates using the 4CL genes from *A. sativa* and *Arabidopsis thaliana* as queries and identified 31 4CL-homologous genes, among them *linA* (DL89DRAFT_225601; e value 1 × 10^–101^) (Table 1). In contrast, when we used the *A. sativa* HHT1 as query, 21 hits with e values of only > 1 × 10^–15^ were obtained. However, when we compared the sequences of the HHT candidates from *L. pennispora* with those from the related lindolin producer *M. pterosporus,* two HHT-like genes [DL89DRAFT_290543 (*linB*) from *L. pennispora* and GQ54DRAFT_300561 from *M. pterosporus*] showed the highest similarity (76% pairwise identity) and lowest bit-score (e value 0) (Table 1). The most likely ligase gene *linA* and the most probable *hht*-like candidate gene *linB* from *L. pennispora* were chosen for expressional profiling. Interestingly, both genes are co-expressed under lindolin producing conditions (Fig. 5A).

**Table 1:** Heterologously expressed genes from *Linderina pennispora* and biochemical function of the corresponding enzymes

Gene	Locus tag	Gene size (bp) (# of introns)	Protein size (aa)	Function	Accession number
*linA*	DL89DRAFT_225601	1841 (2)	553	IAA–CoA ligase (indole-3-acetyl-CoA synthetase)	OR047549
*linB*	DL89DRAFT_290543	1434 (0)	477	indole-3-acetyl-CoA:anthranilate *N*-indole-3-acetyltransferase (lindolin synthase)	OR047550

### LinA is an indole-3-acetate–CoA ligase

The ligase gene *linA* was heterologously expressed in *Escherichia coli*, and the enzyme was purified as a *C*-terminal His_6_-tagged soluble fusion protein. We investigated the substrate specificity of LinA by measuring the released AMP by monitoring the depletion of NADH with a well-established enzyme-coupled assay [49] (Fig. 5B, Additional file 33: Figure S29). LinA preferably uses IAA (**3**) as substrate followed by 2-ketoisovaleric acid, acetate and 2-keto-3-methylvaleric acid (Fig. 5C). The enzyme is highly specific for **3** as it does not convert chemically similar aryl acids such as 4-coumaric acid, caffeic acid or the IAA-analog indole-3-butyric acid (IBA). We determined optimal conditions at pH = 7.5 and ϑ = 35 °C (Additional file 34: Figure S30). Hence, we established LinA as an IAA–CoA ligase.

### LinB is an indole-3-acetyl-CoA:anthranilate *N*-indole-3-acetyltransferase

The transferase LinB was similarly purified from recombinant *E. coli* as an *N-*terminally His_6_-tagged protein (Additional file 33: Figure S29). To characterize LinB, its potential substrate IAA-CoA was synthesized by a reported protocol [50]. When LinB and IAA-CoA were incubated with the acceptor substrates anthranilic acid (AA) or 5-hydroxyanthranilic acid (HAA), formation of **4** or **5** was observed (Fig. 5D). Hence, LinB is sufficient for the production of either lindolin. Moreover, **5** is synthesized from HAA rather than by oxidation of **4**. However, when both anthranilic substrates are equimolarly provided, AA is clearly preferred over HAA (Additional file 35: Figure S31). We determined a pH optimum (pH = 7.8) and a temperature optimum (ϑ = 30 °C) similar to that of LinA (Additional file 36: Figure S32) and assigned LinB as an IAA-CoA:anthranilate *N*-indole-3-acetyltransferase.

### LinA and LinB are sufficient to produce lindolins in vitro

**Fig. 6:**
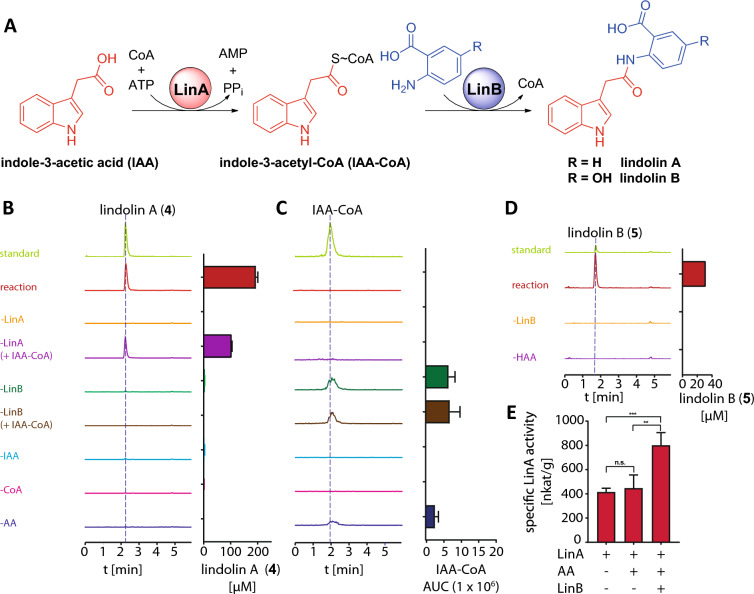
Biocatalytic lindolin production in vitro. **A** Schematic representation of the two-step biosynthesis to lindolins. The reaction was initiated by addition of the substrate anthranilic acid (AA) (**B** and **C**) or 5-hydroxyanthranilic acid (HAA) (**D**) and incubated at 25 °C for 2 h. The amount of **4** (**B**), IAA-CoA (**C**) and **5** (**D**) was chromatographically quantified. Representative EICs are given for **4**, IAA-CoA and **5** at *m/z* 293 [*M*−H]^−^, *m/z* 923 [*M*−H]^−^, and *m/z* 309 [*M*−H]^−^. Note, that the CoA ester leads to slightly broadened peaks as described elsewhere [51]. **E** Enzyme-coupled determination of LinA activity in presence and absence of LinB

We tested the production of lindolins in a one-pot-reaction (Fig. 6A). Indeed, using both enzymes (LinA and LinB), **3**, ATP, CoA and either AA or HAA, the production of **4** or **5**, respectively, was achieved in vitro (Fig. 6B–D). The reaction was strictly dependent on the substrates (IAA, anthranilates), CoA, ATP and both enzymes. A spontaneous amidation was recognized in absence of LinB yielding negligible amounts of lindolins (1%), suggesting that the highly unstable IAA-CoA thioester quenched fast. However, the reaction was highly accelerated by LinB. In addition, an accumulation of IAA-CoA in absence of LinB or its anthranilate substrates was observed (Fig. 6C). In contrast, the intermediate IAA-CoA was scarcely detectable in the coupled enzyme reaction, indicating that LinB converts IAA-CoA as soon as it is produced by LinA. Indeed, LinA’s activity is boosted twofold in the presence of LinB most likely by metabolic channeling of IAA-CoA to LinB (Fig. 6E). In sum, the ligase LinA and the transferase LinB act in concert to produce both lindolins, **4** and **5**.

### The lindolin pathway is of split evolutionary origin

**Fig. 7:**
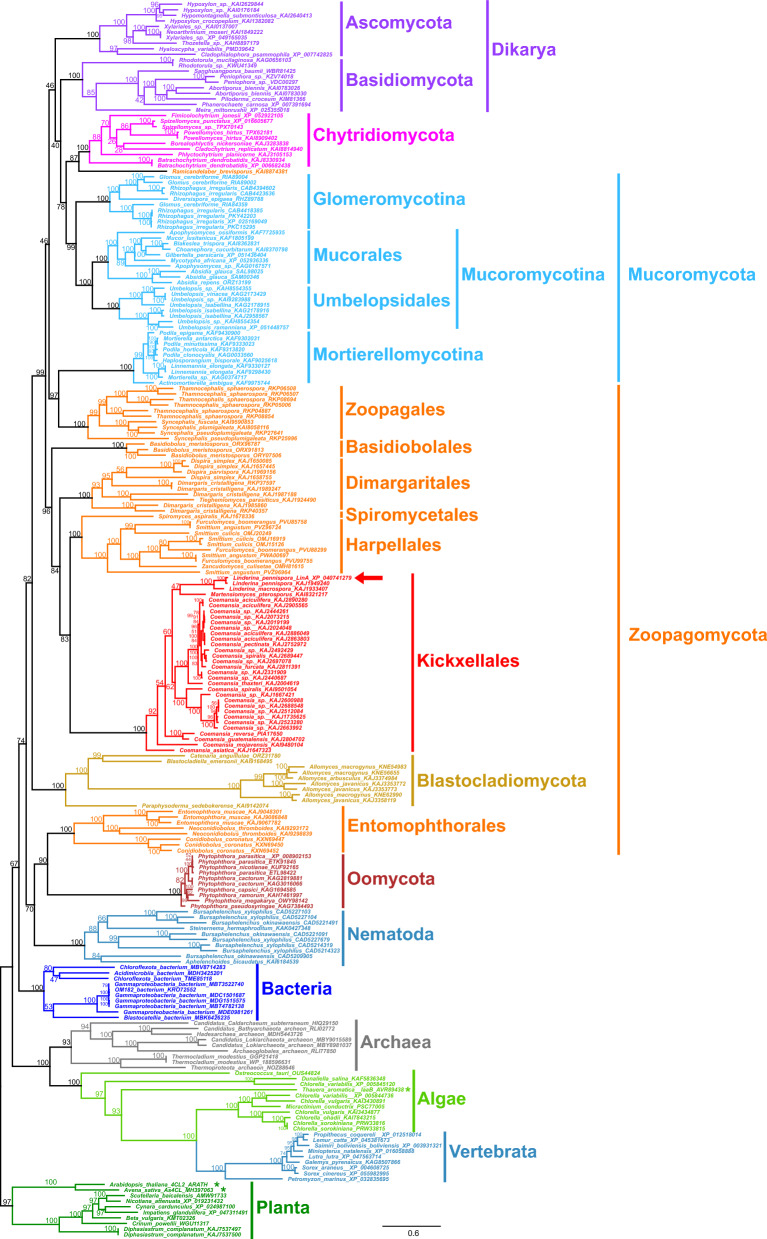
Phylogenetic relationship of LinA and related enzymes in other fungal orders and domains of life. For each phylum or fungal order, ten closest relatives were obtained by BLAST searching the NCBI database. The sequences were aligned using MAFFT and the tree was generated using the maximum-likelihood method of the IQ-tree webserver. All sequences are listed in Additional file 39: Table S8. The tree summarizes 1000 replicates. Plant aryl-CoA synthetases served as outgroup. Bootstrap support values are given for each node (in percent). Asterisks (*) indicate biochemically or genetically verified aryl-CoA ligases. The red arrow highlights the characterized *L. pennispora* LinA

Although *linA* and *linB* are coexpressed, they are not encoded at the same genomic locus neither in *L. pennispora* nor in any other analyzed lindolin producer (Additional file 37: Figure S33, Additional file 38: Figure S34). We hence addressed the potential evolutionary origin of both biosynthetic genes. Phylogenetically, the IAA–CoA ligase LinA from *L. pennispora* clearly clusters with other related enzymes within the order Kickxellales and other orders of Kickxellomycotina such as Harpellales (e value 6 × 10^–131^; pairwise identity 40.04%) and Spiromycetales (1 × 10^–124^; 43.43%) (Table 2, Fig. 7, Additional file 39: Table S8)*.* The enzyme may have evolved from fungal 4-coumaric acid–CoA or ferulic acid–CoA ligases [52] and is only distantly related to well-characterized 4-coumaric acid–CoA ligases (4CL) from plants such as mouse-ear cress (*A. thaliana*) [53], rice (*Oryza sativa*) [54], California poplar (*Populus trichocarpa*) [55]*,* spreading earthmoss (*Physcomitrella patens*) [56] or oat (*A. sativa*) [48]. Moreover, LinA is unrelated to the well-characterized IAA–CoA ligase IaaB from the IAA-degrading bacterium *Aromatoleum aromaticum* [57].

**Table 2:**
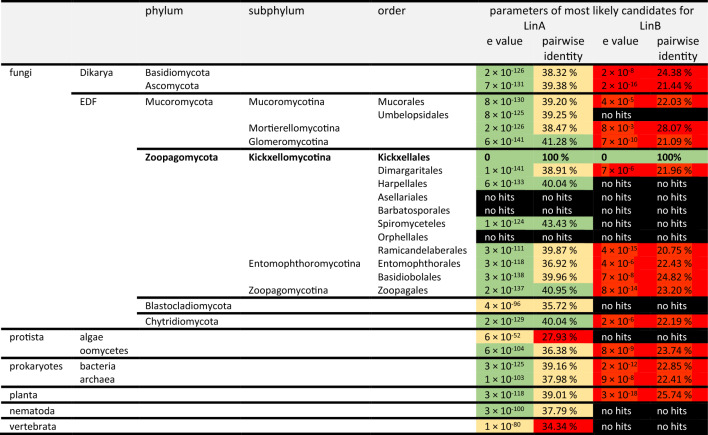
Blast analyses of the closest relatives of the ligase LinA and the transferase LinB

E values of the most likely hits are color-coded (no hits, black; > 10^−20^, red; 10^−20^–10^−100^, yellow; < 10^−100^, green). In addition, pairwise identities of the most likely hits are color-coded (no hits, black; < 35% red, 35–40% yellow, > 40% green)

**Fig. 8:**
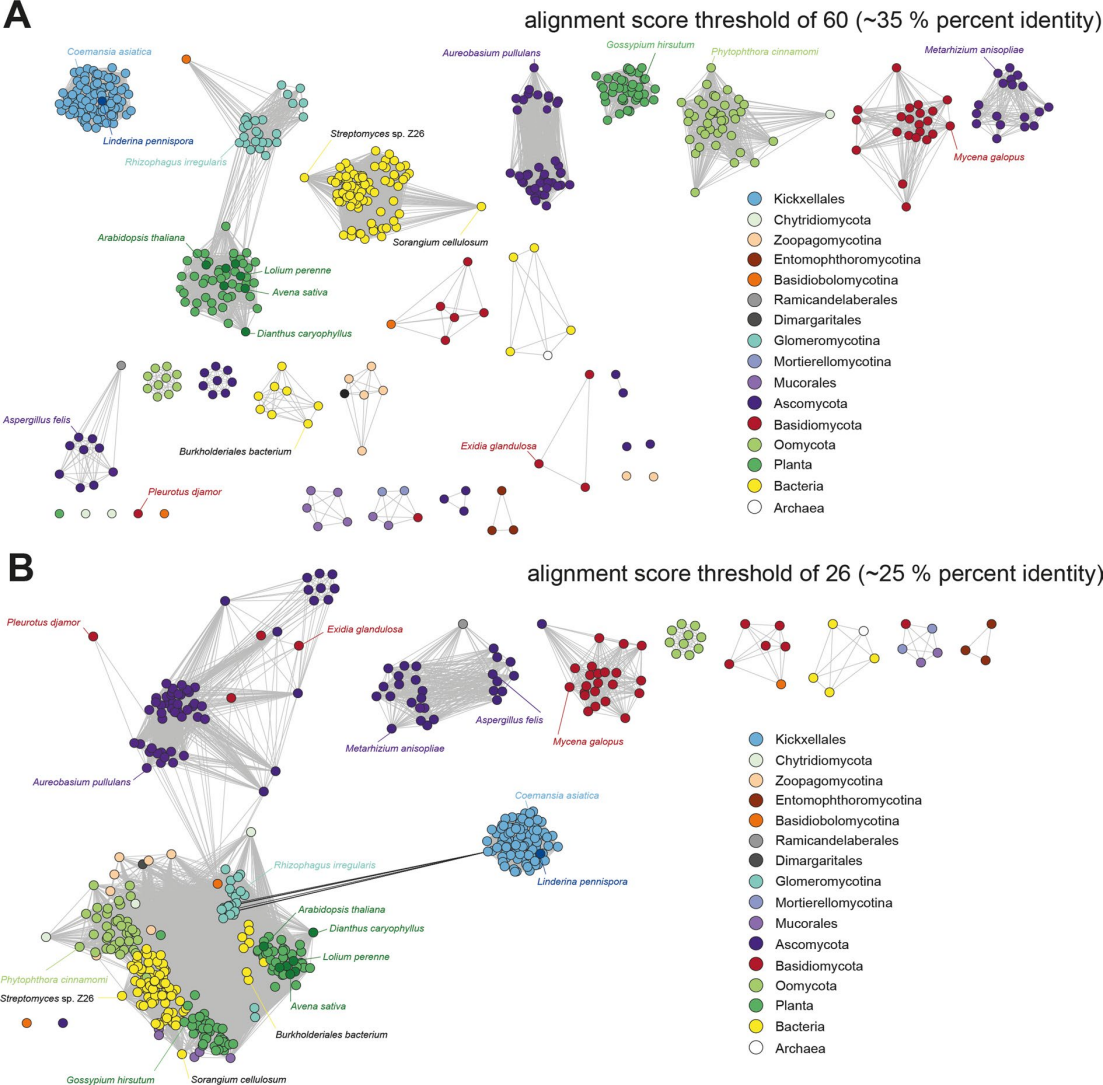
Sequence similarity networks (SSN) for LinB. **A** LinB homologs are depicted in a protein sequence similarity network with an alignment score threshold of 60 (correlating to 35% sequence identity) resulting in a network with distinctly separated clusters corresponding to the different phyla. **B** LinB homologs are depicted in a protein sequence similarity network with an alignment score threshold of 26 (correlating to 25% sequence identity) and showing the sole, but weak correlation between the nodes of LinB sequences from Kickxellales to nodes of other phyla, especially to Glomeromycotina and plants. Each node represents a member of up to 100 closest LinB orthologs in each phylum or fungal order according to the BLAST search (see Additional file 40: Table S9). Nodes are highlighted in color according to the taxonomic classification. Representative examples including LinB from *Linderina pennispora* and biochemically verified LinB-like transferases from plants (*Avena sativa, Arabidopsis thaliana* and *Dianthus caryophyllus*) are indicated in darker color

In contrast, LinB homologs are exclusively found in the genera of Kickxellales such as *Martensiomyces* spp. (e value 0; pairwise identity 61.41%), *Coemansia* spp. (0–5 × 10^–152^; 49.04–63.38%), *Kickxella* spp. (9 × 10^–175^; 51.15%)*,* and *Dipsacomyces* spp. (8 × 10^–172^; 56.86%) (Table 2, Additional file 40: Table S9). LinB is highly specific for this fungal order as there are no closer orthologs present in related Kickxellomycetes, other fungal orders or any eukaryotic or prokaryotic species (> 3 × 10^–18^; < 28.07%) (Table 2). Hence, *linB* must have evolved in the Kickxellales ancestor cell prior to the separation from the other orders and was evolutionary conserved in all descending species. A sequence similarity network (SSN) analysis based on the LinB homologs in Kickxellales and the fairly related proteins from other kingdoms clearly demonstrates the uniqueness of the LinB enzyme class among all kingdoms of life (Fig. 8, Additional file 41: Figure S35, Additional file 42: Figure S36, Additional file 43: Figure S37). Only at a very low alignment score threshold of 26 (at which enzymes of bacteria, plants, Oomycota and Glomeromycotina collapse in a single cluster) a weak correlation between LinB proteins and predicted proteins from Glomeromycotina and plants is detectable. This might point at shikimate-*O*-hydroxycinnamoyl transferases of Glomeromycota or plants as a potential origin of LinB [58]. Indeed, the intensive horizontal gene transfer (HGT) of the shikimate pathway between fungi, plants and prokaryotes is an important driver in eukaryotic genome evolution [59] and HGT has been reported from many EDF [60–62]. From a natural product chemist’s angle, LinB is an evolutionary unique transferase and comprises a non-canonical type of amide-bond forming enzyme in fungal secondary metabolism.

## Discussion

The present work showcases the yet underestimated fungal subphylum Zoopagomycota as potent producers of bioactive natural compounds. Several species of the fungal order Kickxellales produce the IAA-derived indole amide anthranilates lindolin A and B, which possess anti-oomycete activity. The unusual amidation is NRPS-independent, but coenzyme A-mediated and relies on the activity of two enzymes, the ligase LinA and the unique transferase LinB.

The production of IAA (**3**) and its derivatives such as tryptamine (TAM), indole-3-acetamide (IAM), ILA (**1**), indole-3-pyruvic acid (IPyA), IOL (**2**), and indole-3-acetonitrile (IAN) is a widely distributed feature of plant-interacting fungi, especially for ectomycorrhizal basidiomycetes such as the “skin mushroom” *Astraeus odoratus* [42], the alder bolete *Gyrodon lividus* [42], and the scaly knight (fuzztop) *Tricholoma vaccinum* [41]. However, **3** amides have not been described for fungi yet. **3** amide conjugates were primarily thought of as storage form of auxin in plants [63], but were later recognized to regulate seedling development, **3** homeostasis and abscission [64–66]. Our study did not verify auxinic or phytotoxic effects of **3**-derived lindolins, but identified them as anti-oomycetic agents. Oomycetes are mainly filamentous, heterotrophic species closely related to diatoms and brown algae [67] and include important phytopathogens such as the aggressive cosmopolitan necrotroph *Pythium* spp. and the host-specific hemibiotroph *Phytophthora* spp. [68]. Both cause blights in agricultural important hosts such as potato (*Solanum tuberosum*), tomato (*Solanum lycopersicum*), or soybean (*Glycine max*) [68]. Oomycetes are hardly susceptible for many antifungal drugs (especially azoles) as they possess a plant-like cell wall and membrane composition [69]. Effective concentrations of anti-oomycete drugs are usually higher than used for antibiotics or antimycotics. For example, the anti-oomycete compound 2*E*,4*E*-decadienoic acid (DDA) identified from *Trichoderma asperellum* [70] is active against *Phytophthora* spp. at 594 µM (100 µg mL^−1^). However, synthetic antifungals such as azoxystrobin (5 µM; 2 µg mL^−1^) and metlaxyl (54 µM; 15 µg mL^−1^) [71] or oocydin isolated from *Serratia marcescens* (63 nM; 0.03 µg mL^−1^) [72] are active against oomycetes at much lower concentrations. Despite the high MIC_50_ of **4** (100 µM; 34 µg mL^−1^), inhibitory titers are readily reached by *L. pennispora* due to high production rates of up to 500 µM.

Anthranilate moieties are common in bacterial benzoxazole antibiotics including nataxazole [73], carboxamycin [74], calcimycin [75] and A33853 [76] as well as in many fungal nonribosomal peptides including acetylaszonalenin, fumiquinazoline A and asperlicin [77]. Benzoxazole antibiotics show diverse biological properties including anticancer and antibacterial activities [78]. Anthranilate is commonly derived from l-tryptophan degradation, whilst 5-hydroxyanthanilate might be produced by an anthranilate-5-hydroxylase during anthranilate catabolism [79–81]. Recently, a flavin-dependent monooxygenase Aha6 (UMM61384.1) was predicted to convert anthranilate into its 5-hydroxy derivative during tasikamide biosynthesis in *Streptomyces tasikensis* [82]. However, none of the lindolin producers encodes a *aha6* homolog and 5-hydroxyanthranilate might be produced by alternative shikimate anabolic or l-tryptophan catabolic pathways [83].

**Fig. 9:**
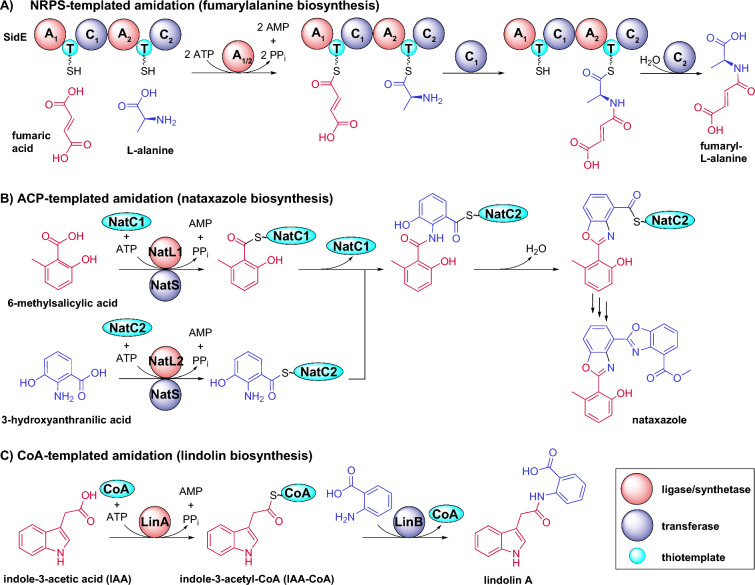
Thiotemplated amidation reactions in microbes. The thiotemplated amidation in secondary metabolism requires an active thioester prior to peptide transfer. The donor acid is usually bound to a carrier protein (CP) that is either part of an NRPS (**A**) or a freestanding CP domain protein (**B**). In contrast, lindolin biosynthesis in Kickxellomycetes rely on a CP-independent but CoA-mediated amide formation (**C**). *A* adenylation domain, *ACP* acyl carrier protein, *C* condensation domain, *NatC1/2* freestanding CPs, *NatL1/2* aryl-AMP synthetases, *NatS* aryl-ACP synthases, *T* thiolation domain (NRPS-bound CP). Donor and acceptor acids are highlighted in blue and red, respectively

The final step in lindolin biosynthesis is the LinA/LinB-catalyzed condensation of **3** with anthranilate. ATP-dependent amide bond formation of small molecules is widely distributed in nature and is mainly catalyzed by ribosomes [84], NRPSs [85] and CoA-dependent acyl transfer systems especially during siderophore metabolism [86] and histone modification [87]. In the microbial secondary metabolism, the thiotemplated amidation usually requires the tethering of the donor amino acid to a thiolated carrier protein (CP) [88]. CPs are either embedded in an NRPS as so-called thiolation (T) domains as exemplified in the fumaryl-l-alanine biosynthesis [39] (Fig. 9A) or act as small, stand-alone CP domain proteins as shown for the nataxazole biosynthesis [73] (Fig. 9B). Kickxellomycetes follow a third, CP-independent route for thiotemplated peptide formation by using a plant-like CoA-mediated catalysis mechanism (Fig. 9C). Interestingly, isolated adenylation (A) domains of NRPSs can close peptide bonds in vitro as well [89–91]. Similar to the native LinA/LinB-coupled system, thioester-mediated biocatalytic syntheses of dozens of different dipeptides have been demonstrated in a one-pot-reaction using two promiscuous enzymes, the A domain of the carboxylic acid reductase CAR*sr* coupled to the plant-derived tyramine-*N*-hydroxycinnamoyl acyltransferase *Ca*AT [91]. Using this approach, even post-translational modifications of proteins can be achieved. This result highlights the CoA-mediated amide formation as a powerful strategy to label or click-functionalize proteins.

The non-canonical biosynthesis of lindolins show a remarkable similarity to the structurally related 5-hydroxyanthranilate amides avenanthramides A–C (formerly known as avenalumins) from oat [48, 92, 93]. However, the biosynthetic genes are only marginally related and may have independently evolved. Avenanthramides were initially discovered as defensive phytoalexins against the crown rust *Puccinia coronata* [94] but were later reidentified in oat grains [95]. Avenanthramides inhibit atherosclerosis and asthma by suppression of both inflammatory cytokines and adhesion molecules in endothelial cells [96]. Since 1982, their structural derivative tranilast (brand name: rizaben) has been marketed in Asia to treat allergic disorders [97] and is additionally a proven chemotherapeutic drug in several pre-clinical studies [98]. In contrast, lindolins do not show antiproliferative effects on mammalian cell lines. However, a more profound investigation of their function during inflammation might be an objective of future works.

Tremendous effort has been conducted by comparative genomics of hundreds of EDF genomes to identify novel biosynthetic gene clusters [1, 2, 27]. Using this promising approach, potential SM producers including the genera *Mortierella* [12, 37]*, Mucor* [99]*, Phycomyces* [100], and *Basidiobolus* [21, 22] were identified. However, previous works mainly relied on the identification of well-known SM key enzymes (e.g. NRPSs, polyketide synthases, terpene synthases and hybrids thereof) encoded within a genetic cluster, but neglected the identification of non-canonical biosynthesis pathways or non-clustered genes. Both features apply to the lindolin biosynthesis in Kickxellales. With a few exceptions, cooperatively acting SM genes are usually co-expressed and co-located in biosynthetic gene clusters in both Ascomycota [101, 102] and Basidiomycota [103–105]. Opposingly, lindolin biosynthetic genes *linA* and *linB* are co-expressed, but not clustered. Similarly, the production of the β-carotene-derived Mucorales mating hormone trisporic acid requires several non-clustered enzymes [106–108] suggesting that SM biosynthetic genes might be not clustered in EDF in general—a phenomenon that is widely known in plant secondary metabolism [109]. Pharmacologically important non-canonical pathways include the biosynthesis of the aforementioned anti-inflammatory avenanthramides [48], the neuroactive drug psilocybin [103], and the fly agaric toxin ibotenic acid [104], and is now expanded by the anti-oomycete lindolins from the yet underestimated fungal order Kickxellales.

Our study shows that the current strategies to identify biosynthetic routes in EDF need to be re-evaluated and the archetypical “gene cluster concept” might be reconsidered for EDF. Moreover, the non-clustered lindolin biosynthesis revealed that the metabolic potential of EDF is manifolded and much more diverse than previously predicted by genomic approaches.

## Conclusion

Kickxellomycetes are a novel promising source of bioactive natural products including indole alkaloids. The non-canonical, NRPS-independent biosynthesis of the indole alkaloids lindolin A and B relies on two separate enzymes that are not encoded in a biosynthetic gene cluster. This may affect future genetic studies on secondary metabolism in EDF in general. Moreover, the metabolic potential of EDF is larger than previously recognized pointing to an underestimated reservoir for natural compounds.

## Methods

### Organisms and culture conditions

All fungal EDF strains were initially verified by ITS sequencing [10] (Additional file 1: Table S1) and were routinely cultivated on MEP agar plates [20 g L^−1^ malt extract (Carl Roth), 3 g L^−1^ soy peptone (Gibco), 20 g L^−1^ agar, pH 5.6] for 7–14 days at 25 °C (*Coemansia* spp.) or 30 °C (*Linderina* sp. and *Martensiomyces* sp.). The mycoparasite *Dimargaris bacillispora* CBS218.59 was grown on its fungal host *Cokeromyces recurvatus* on V8 agar [100 mL L^−1^ V8 juice (Campbell Soup), 0.75 g L^−1^ CaCO_3_, 15 g L^−1^ agar] at 25 °C for 14 days. To induce lindolin biosynthesis, 100 mL meat medium [10 g L^−1^ meat extract (Carl Roth), 10 g L^−1^ tryptone (Carl Roth), 5 g L^−1^ NaCl], MEP medium, or PDB [26.5 g L^−1^ potato dextrose broth (Carl Roth)] were inoculated with three agar blocks (9 mm^2^) of the respective strains and incubated at 160 rpm at 25 °C or 30 °C for 3 to 21 days. Cultures were additionally grown on meat medium, MEP and PDB agar plates containing 20 g L^−1^ agar for 7–28 days at 25 °C or 30 °C. To show l-tryptophan-dependent production of **3**–**5**, *L. pennispora* was cultivated on *Aspergillus* minimal medium (AMM) [110] without d-glucose but with 1% (w/v) casamino acids (Difco) and with or without the supplementation of 5 mM l-tryptophan, for three days. *L. pennispora*, *M. pterosporus* and *C. furcata* were cultivated emersly on 20 g autoclaved potting soil (“Floralie” from Floraself) supplemented with or without 1% (w/w) d-glucose for ten days. Oomycete strains (*P. megasperma* and *P. macrosporum*) were verified by ITS sequencing [10] (Additional file 1: Table S1) and were grown on PDB agar at 25 °C for up to seven days. *F. graminearum* was cultivated on MEP agar at 30 °C for 4 days. *Escherichia coli* XL1 blue was used for plasmid propagation and *E. coli* SoluBL21 (for LinA) and BL21 (for LinB) were used for protein production (Additional file 1: Table S1). *E. coli* was cultivated at 37 °C on LB agar plates supplemented with 100 µg mL^−1^ carbenicillin (Roth) or 50 µg mL^−1^ kanamycin (Roth), if required.

### Chromatographic analysis of metabolite extracts

#### Extraction of metabolites from culture filtrate, mycelium, agar plates and soil

Culture filtrates were separated from the mycelium using Miracloth (Merck). The mycelium was rinsed with water, lyophilized to dryness, weighed to determine the fungal dry biomass and extracted with 5 mL methanol per 100 mg dry fungal biomass. The culture filtrates were adjusted to pH 6 by addition of 3 M HCl or 3 M NaOH prior to extraction with 100 mL ethyl acetate. Cultivated agar plates (25 mL) were sliced in 5 × 5 mm pieces and extracted overnight with 25 mL ethyl acetate. 20 g of soil samples were extracted with 40 mL of ethyl acetate. In all extraction procedures, uninoculated liquid medium, agar or soil served as negative control. The organic phase was collected and evaporated to dryness using a rotary evaporator. The residue was dissolved in 2 mL methanol, ultrasonicated for 1 min, and centrifuged to remove contaminating particles (20,000×*g*, 10 min). 5 µL thereof were used for GC–MS and UHPLC measurements (methods 1 and 2, Additional file 44: Table S5).

#### GC–MS analysis

The compounds ILA (**1**), IOL (**2**) and IAA (**3**) were determined by GC–MS on a Trace 1310 gas chromatograph (Thermo Fisher Scientific) coupled with a TSQ 9000 electron impact (EI)-triple quadrupole mass spectrometer using method 1 (Additional file 44: Table S5). A 4 mm SSL GC inlet glass liner with glass wool (P/N 453A1305) and a BPX5 capillary column (30 m, 0.25 mm inner diameter, 0.25 μm film) from Trajan (SGE) was used. The column was operated with helium carrier gas (1.5 mL min^−1^) and split injection (split ratio 1:10). Total ion current (TIC) values were recorded in the mass range of 45–500 amu, with a scan time of 0.2 s. and a MS delay of 4 min. As **1** and **3** were too hydrophilic for optimal GC runs, silylation of **1–3** was conducted with *N*-methyl-*N*-(trimethylsilyl) trifluoroacetamide (MSTFA) prior to GC analysis: Methanolic fungal extracts were dried *in vacuo* and resuspended in 150 µL MSTFA. Samples were mixed for 15 s, ultrasonicated for 1 min and centrifuged. A volume of 1 µL of the supernatant was injected. The National Institute of Standards and Technology (NIST) Mass Spectra Search Program version 2.4 was used for comparison of the EI-MS spectra.

#### UHPLC-MS analysis

Compounds **3**, **4** and **5** as well as IAA-CoA were analyzed on an Agilent 1290 Infinity II UHPLC instrument, coupled to a 6130 single quadrupole mass spectrometer using method 2 (Additional file 44: Table S5). Metabolite quantification was carried out using a regression curve based on the injection of gradual binary dilutions of **3**, **4** and **5** from 3 to 800 µM.

### Purification and structure elucidation of lindolin A (4) and B (5)

*L. pennispora* was grown in an upscaled culture of 12 L AMM with 1% (w/v) casamino acids and 5 mM l-tryptophan with orbital shaking at 160 rpm at 30 °C for 10 days. Metabolites were extracted from the supernatant as described above. The residue was dissolved in 130 mL dichloromethane and reduced to 40 mL with a rotary evaporator. Purification of **4** and **5** was conducted using a Büchi C-810 Flash Chromatograph and an Agilent 1200 Infinity HPLC system equipped with a diode array detector (DAD). The extract was subjected to a normal phase column (Silica gel, 40 g) on the flash chromatograph using method 3 (Additional file 44: Table S5). Four fractions (fraction I–IV) were obtained, of which fractions II and III contained **5** and **4**, respectively.

Fraction III (containing **4**) was evaporated to dryness and dissolved in 3 mL methanol. In four individual runs, 750 µL thereof were flash-chromatographed using a reverse phase column (C18, 12 g) running method 4 (Additional file 44: Table S5). The obtained fraction with the major peak of **4** was again dried by lyophilization and dissolved in 2 mL methanol. In several runs, aliquots of 10 µL each were subjected to semipreparative HPLC purification using method 5 (Additional file 44: Table S5). A total of 40 mg of pure** 4** were obtained. Fraction II (containing **5**) was evaporated to dryness and dissolved in 3.5 mL methanol. Purification was accomplished on a reverse phase column (12 g, C18) using method 6 (Additional file 44: Table S5) followed by semipreparative HPLC using method 7 (Additional file 44: Table S5). A total of 14 mg of pure** 5** were obtained. MS/MS measurements were performed using a Q Exactive Plus mass spectrometer (Thermo Scientific). NMR spectra were recorded on a Bruker Avance III 600 MHz spectrometer at 300 K. DMSO-*d*_6_ served as solvent and internal standard (δ_H_ = 2.50 ppm and δ_C_ = 39.52 ppm).

### Synthesis of chemical standards

Indole-3-ethanol (IOL, **2**) was synthesized by reduction of IAA (**3**) with lithium aluminium hydride in THF according to Du et al*.* [111]. Indole-3-acetyl coenzyme A (IAA-CoA) was synthesized by thioesterification of IAA (**3**) with coenzyme A trilithium salt (Merck) according to Pourmasoumi et al*.* [50].

### Biological activities

Antimicrobial activities were initially determined at a concentration of 1 mg mL^−1^ methanol of **4** and **5** against the following strains according to a well-established protocol [38]: *Bacillus subtilis* ATCC6633, *Staphylococcus aureus* SG511, *Escherichia coli* DSM498, *Pseudomonas aeruginosa* K799/61, *Mycobacterium vaccae* IMET10670, *Sporidiobolus salmonicolor* SBUG0549, *Candida albicans* JMRC:STI:50163 and *Penicillium notatum* JMRC:STI:50164. Anti-oomycete activities were determined by cultivation of *Phytophthora megasperma* CBS 687.79 and *Pythium macrosporum* CBS 575.80 on PDB agar plates in presence of serial binary dilutions of lindolin A (**4**) and B (**5**) (7.8–500 µM) for 4–6 days measuring the colony diameter. Pure solvent (methanol) or serial dilutions of hygromycin B (10–400 µM) served as negative and positive controls, respectively. Antiproliferative or cytotoxic activities were determined for human umbilical vein endothelial cells (HUVEC) or cervical cancer cells (HeLa), respectively, as described [10]. Growth-inhibitory and auxin-inhibitory activity of **4** and **5** was calculated on radish seedlings (Kiepenkerl) according to a published protocol [112, 113]. In brief, radish seeds were cultivated in Hoagland medium agar [112] supplemented with 1 and 10 µM of **4** and **5** (growth-inhibitory effect). Solvent (methanol) and **3** (1 µM) were negative and positive controls, respectively. A competitive assay using 1 µM **3** and 1 µM **4** or **5** was carried out similarly (auxin-inhibitory effect). Root and hypocotyl growth were measured daily for a period of 5 days.

### Isolation of nucleic acids and expression analysis

Fungal mycelium from 36 h cultures in AMM, PDB and meat medium was lysed with glass beads (1–5 mm) in a FastPrep Homogenizer (MP Bio) for 2 min at 4.5 m s^−1^. Genomic DNA was isolated as described [38]. RNA was isolated with the SV Total RNA Isolation System (Promega) using the manufacturer’s protocol. RNA (1 µg) was DNase-treated (Baseline-ZERO, Lucigen) and was reversely transcribed into cDNA by the RevertAid RT kit (Thermo) using anchored oligo-(dT)_20_ oligonucelotides. Expression analysis was performed at the AnalytikJena qTower^3^ using the qPCR Mix EvaGreen (Bio&SELL) and oligonucleotides with a minimum primer efficiency of 95% (Additional file 45: Table S6). After an initial denaturation at 95 °C for 15 min, 40 cycles of amplification were run (95 °C, 15 s; 60 °C, 20 s; 72 °C, 20 s). The housekeeping reference genes encoding actin (*actA*, DL89DRAFT_257372), the TEF transcription factor (*tefA*, DL89DRAFT_11075) and the glyceraldehyde-3-phosphate dehydrogenase (*gpdA,* DL89DRAFT_277503) served as internal standards. Gene expression levels were determined as described by Pfaffl [114].

### Enzymatic assays

#### Heterologous protein production

*linA* and *linB* were amplified from *L. pennispora* cDNA using Phusion High-Fidelity DNA polymerase (NEB) and the oligonucleotides listed in Additional file 45: Table S6. The fragments were ligated into the pET28a expression vector using the restriction sites *Nco*I/*Hind*III and *Nhe*I/*EcoR*I (Additional file 46: Table S7), respectively. Expression was conducted in *Escherichia coli* SoluBL21 (*linA*) and BL21 (*linB*), cultivated in 400 mL LB medium supplemented with 50 µg mL^−1^ kanamycin at 37 °C until an optical density of 0.6 was reached. Then, expression was initiated by adding 1 mM IPTG. After another 16 h of incubation at 16 °C, cells were harvested by centrifugation (4000×*g*, 25 min) and disrupted in 5 mL lysis buffer (50 mM sodium phosphate, 300 mM NaCl, 10 mM imidazole, pH 8) by ultrasonication. The enzymes were purified using the Protino Ni^2+^ NTA Agarose (Macharey & Nagel) according to the manufacturer’s protocol by a step gradient (10–250 mM imidazole). The proteins were subsequently concentrated and re-buffered in assay buffer (200 mM Tris, pH 7.8) using the Amicon Ultra-15 Central Filter System (30 kDa cut-off). The protein concentration was determined by the Pierce BCA protein Assay (Thermo Fisher Scientific) using albumin as reference standard. The purity was additionally verified by SDS polyacrylamide gel electrophoresis (Additional file 33: Figure S29). The enzymes were stored at 4 °C without loss of activity for up to three weeks.

#### Determination of LinA activity

To determine the substrate specificity of LinA in a 200 µL scale, a modified coupled enzyme assay with myokinase, pyruvate kinase and lactate dehydrogenase according to Patel et al. was used [49, 115]. Final concentrations were: 200 mM Tris (pH 7.8), 20 mM MgCl_2_, 360 µM NADH, 1 mM phosphoenolpyruvate, 2.5 mM ATP, 250 µM coenzyme A, 1 U mL^−1^ myokinase (Merck), 1 U mL^−1^ pyruvate kinase (Merck), 3 U mL^−1^ lactate dehydrogenase (Merck) and 1 µM LinA. The reaction was initiated with 1 mM of substrates (small carboxy acids including IAA, see Fig. 5, all from Merck). Water was used as negative control. The continuous assay relies on the NADH consumption which is photometrically detected at λ = 340 nm using the ClarioStar microplate reader at 30 °C for up to 30 min.

#### Determination of LinB activity

To determine LinB activity, a discontinuous UHPLC-based assay was used. The final assay (500 µL) contained 200 mM Tris (pH 7.8), 20 mM MgCl_2_, 1 mM IAA-CoA and 1 µM LinB. The reaction was started with either 1 mM anthranilate (AA) or 5-hydroxyanthranilate (HAA) (Merck). Water served as negative control. After 2 h of incubation at 25 °C, the reaction was stopped by shock freezing in liquid nitrogen and lyophilization. The residue was dissolved in an equal volume acetonitrile: water (50: 50), briefly centrifuged (14,000×*g*, 2 min) and immediately subjected to UHPLC analysis using method 2 (Additional file 44: Table S5). A calibration curve of **4**,** 5** and IAA-CoA was recorded as standard for quantification.

#### Combined LinA/LinB assay

To produce **4** and **5** in vitro, the following assay was set up in a 500 µL scale: 200 mM Tris (pH 7.8), 20 mM MgCl_2_, 250 µM CoA, 2.5 mM ATP, 1 µM LinA, 1 µM LinB, and 1 mM AA and/or HAA. As controls, individual assay components were omitted. The reaction was initiated by addition of 1 mM IAA (**3**). The assay was incubated for 120 min at 25 °C. Lindolin detection was carried out by UHPLC-MS measurements as described above.

### Bioinformatic analyses

#### Gene cluster analysis

Comparative gene cluster analysis was performed with CAGECAT [116], an online comparative gene cluster analysis toolbox which allows homology searches between whole gene clusters or regions. The implemented clinker tool [117] was used to visualize the alignment of the genomic neighborhood of the *linA* and *linB* gene loci (± 20 kb) extracted from the genomes of eight verified lindolin-producing Kickxellales species. The minimum alignment sequence identity was set to 30%.

#### Phylogenetic analysis of LinA

LinA-related enzymes were identified using the BLAST search tool (matrix: BLOSUM62; gap costs: existence 11, extension 1; expect threshold: 0.05) screening the non-redundant protein sequences (nr) of NCBI for each fungal order/phylum and other domains of life separately. For each order or phylum, ten (if available) most-likely hits (according to bit score) were depicted for further analysis. Sequences with less than < 400 or > 700 amino acids were omitted as *L. pennispora* LinA comprises 553 aa. Moreover, biochemically verified aryl-CoA ligases were additionally included [48, 53, 57]. Protein accession numbers, e values and pairwise identities are listed in Additional file 39: Table S8. A total of 222 amino acid sequences were aligned using MAFFT online [118] (matrix: BLOSUM62; gap open penalty: 1.53; gap extension penalty: 0.123; tree rebuilding number: 50; max iteration number: 100). The alignment was extracted to the IQ-Tree webserver [119] to generate Maximum-Likelihood trees with LG + R8 as best-fit model and a total of 1000 replicates (bootstrap support). Single branch tests were performed by SH-aLRT. A bootstrap consensus tree was computed and included 222 taxa with 763 splits.

#### Construction of sequence similarity networks (SSN) for LinB

LinB-related enzymes were identified using the BLAST search tool (matrix: BLOSUM62; gap costs: existence 11, extension 1; expect threshold: 0.05) screening the non-redundant protein sequences (nr) of NCBI for each fungal order/phylum and other domains of life separately. For each order or phylum, 100 (if available) most-likely hits (according to bit score) were depicted for further analysis. Moreover, biochemically verified aryl-transferases were additionally included [48, 58, 120]. Protein accession numbers, e values and pairwise identities are listed in Additional file 40: Table S9. SSNs were constructed using the Enzyme Function Initiative-Enzyme Similarity Tool (EFI-EST) [121]. Two different SSNs were generated with EFI-EST using the default setting of a −log(e value) = 5 for all-by-all BLAST to calculate similarities and edge alignment score similarities resulting in 505 nodes and > 25,000 edges. The sequences in the generated SSN were restricted to a sequence length of 400–600 amino acids (Additional file 41: Figure S35, Additional file 42: Figure S36, Additional file 43: Figure S37). The alignment score threshold was set to 60 (corresponding to 35% sequence identity) or 26 (25%) for figure panels 8A and 8B, respectively. Data was visualized in Cytoscape v. 3.10.2 [122] using a “prefuse force directed layout” with the option “all nodes: alignment_score”.

### Supplementary Information

**Additional file 1: Table S1.** Microbial strains.

**Additional file 2: Figure S1.** Atom numbering and selected COSY (bold lines) and HMBC (red arrows) in **4**.

**Additional file 3: Figure S2.** ^1^H NMR spectrum of **4** in DMSO-*d*_6_.

**Additional file 4: Figure S3.** ^13^C NMR spectrum of** 4** in DMSO-*d*_6_.

**Additional file 5: Figure S4.** DEPT-135 NMR spectrum of **4** in DMSO-*d*_6_.

**Additional file 6: Figure S5.** ^1^H-^1^H COSY spectrum of **4** in DMSO-*d*_6_.

**Additional file 7: Figure S6.** ^1^H-^13^C HSQC spectrum of **4 **in DMSO-*d*_6_.

**Additional file 8: Figure S7.** ^1^H-^13^C HMBC spectrum of **4** in DMSO-*d*_6_.

**Additional file 9: Figure S8.** ^1^H-^1^H TOCSY spectrum of **4** in DMSO-*d*_6_.

**Additional file 10: Figure S9.** ESI-MS/MS spectrum of **4**.

**Additional file 11: Table S2.** NMR data of **4** in DMSO-*d*_6_.

**Additional file 12: Figure S10.** Atom numbering and selected COSY (bold lines) and HMBC (red arrows) in **5**.

**Additional file 13: Figure S11.** ^1^H NMR spectrum of **5** in DMSO-*d*_6_.

**Additional file 14: Figure S12.** ^13^C NMR spectrum of **5** in DMSO-*d*_6_.

**Additional file 15: Figure S13.** DEPT-135 NMR spectrum of **5** in DMSO-*d*_6_.

**Additional file 16: Figure S14.** ^1^H-^1^H COSY spectrum of **5** in DMSO-*d*_6_.

**Additional file 17: Figure S15.** ^1^H-^13^C HSQC spectrum of **5** in DMSO-*d*_6_.

**Additional file 18: Figure S16.** ^1^H-^13^C HMBC spectrum of **5** in DMSO-*d*_6_.

**Additional file 19: Figure S17.** ^1^H-^1^H TOCSY spectrum of **5** in DMSO-*d*_6_.

**Additional file 20: Figure S18.** ESI-MS/MS spectrum of **5**.

**Additional file 21: Table S3.** NMR data of **5** in DMSO-*d*_6_.

**Additional file 22: Figure S19.** GC-MS/MS spectrum of ILA (**1**).

**Additional file 23: Figure S20.** GC-MS/MS spectrum of IOL (**2**).

**Additional file 24: Figure S21.** GC-MS/MS spectrum of IAA (**3**).

**Additional file 25: Figure S22.** Production of lindolin A and B in Kickxellales and related species.

**Additional file 26: Figure S23.** Antimicrobial activities of **4** and **5**.

**Additional file 27: Figure S24.** Antiproliferative and cytotoxic activities of **4** and **5** against mammalian cells.

**Additional file 28: Figure S25.** Plant growth activating and IAA-interfering properties of IAA (**3**), lindolin A (**4**) and lindolin B (**5**) on radish seedlings.

**Additional file 29: Figure S26.** Initial screening for antifungal and anti-oomycete properties of lindolin A (**4**).

**Additional file 30: Figure S27.** Determination of the minimal inhibitory concentration (MIC_50_) of lindolin A (**4**) on oomycetes.

**Additional file 31: Table S4.** BLAST search for homologous genes required for IAA production in Kickxellales.

**Additional file 32: Figure S28.** Quantification of IAA (**3**), lindolin A (**4**) and lindolin (**5**) in cultures of *L. pennispora*.

**Additional file 33: Figure S29.** SDS polyacrylamid gel electrophoresis (SDS-PAGE) of purified His_6_-tagged LinA and LinB.

**Additional file 34: Figure S30.** Determination of the optimal reaction conditions for LinA.

**Additional file 35: Figure S31.** Activity of LinB during competitive substrate supply.

**Additional file 36: Figure S32.** Determination of the optimal reaction conditions for LinB.

**Additional file 37: Figure S33.** Clinker comparison between *linA *gene loci in Kickxellales.

**Additional file 38: Figure S34.** Clinker comparison between *linB *gene loci in Kickxellales.

**Additional file 39: Table S8.** BlastP analysis of LinA-like proteins in species of various kingdoms of life.

**Additional file 40: Table S9.** BlastP analysis of LinB-like proteins in species of various kingdoms of life.

**Additional file 41: Figure S35.** Alignment lengths of LinB-like proteins in SSN.

**Additional file 42: Figure S36.** Percent identity depending on the alignment score of LinB-like proteins in SSN.

**Additional file 43: Figure S37.** Histogram of sequence lengths of LinB-like proteins used for SSN analysis.

**Additional file 44: Table S5.** Chromatographic Methods.

**Additional file 45: Table S6.** Oligonucleotides used in this study.

**Additional file 46: Table S7.** Plasmids used in this study.

## Data Availability

The sequences of the *linA* and *linB* genes from *L. pennispora* were deposited under the GenBank accession numbers OR047549 and OR047550. The ITS sequences of the EDF strains were deposited at Genbank according to Additional file 1: Table S1. The fungal strains are available upon request from the Jena Microbial Resource Collection (JMRC), the Westerdijk Fungal Diversity Institute or the American Type Culture Collection (ATCC) as listed in Additional file 1: Table S1.
