# Supplementary material for: Non-canonical two-step biosynthesis of anti-oomycete indole alkaloids in Kickxellales
Source: Fungal Biol Biotechnol. 2023 Sep 5;10:19. doi: 10.1186/s40694-023-00166-x (PMC10478498; doi:10.1186/s40694-023-00166-x)
Supplement: Supplementary file 1 — Additional file 1: Table S1. Microbial strains. [file 40694_2023_166_MOESM1_ESM.pdf]

Table S1. Microbial strains. CBS, Westerdijk Fungal Biodiversity Institute; FGSC, Fungal Genetics Stock Center; JMRC, Jena Microbial resource collection.

organism	phylum - order	strain number (JMRC)	other strain numbers	lindolin production	ITS accession number	reference
Linderina pennispora	Zoopagomycota - Kickxellales	JMRC:SF:000819	ATCC12442<br>CBS 312.51<br>NRRL2237<br>VKM:F1219	yes	OR045897	JMRC
Martensiomyces pterosporus	Zoopagomycota - Kickxellales	JMRC:SF:000821	CBS 209.56<br>IMI 060573	yes	OR045898	JMRC
Coemansia furcata	Zoopagomycota - Kickxellales	JMRC:SF:033834	DSM6934<br>S005<br>CRC 31803	yes	OR045893	JMRC
Coemansia aciculifera	Zoopagomycota - Kickxellales	JMRC:SF:002823	ZM 17	yes	OR045891	JMRC
Coemansia asiatica	Zoopagomycota - Kickxellales	JMRC:SF:000795	CBS 336.87<br>FMR 845	yes	OR045892	JMRC
Coemansia mojavensis	Zoopagomycota - Kickxellales	JMRC:SF:011250	CBS 166.58	(yes)	OR045895	JMRC
Coemansia guatemalensis	Zoopagomycota - Kickxellales	JMRC:SF:002937	BI 104	yes	OR045894	JMRC
Coemansia pectinata	Zoopagomycota - Kickxellales	JMRC:SF:002700		yes	OR045896	JMRC
Dimargaris bacillispora	Zoopagomycota - Dimargaritales		CBS 218.59<br>IMI 130774<br>RSA 592	no	-	CBS
Fusarium graminearum	Ascomycota - Hypocreales	JMRC:SF:005402		no	-	JMRC
Aspergillus nidulans	Ascomycota - Eurotiales		FGSC A4	no	-	FGSC
Phytophthora megasperma	Oomycota - Peronosporales	JMRC:SF:002548	CBS 687.79	no	OR045899	JMRC
Pythium macrosporum	Oomycota - Peronosporales	JMRC:SF:002543	CBS 575.80	no	OR045900	JMRC
Escherichia coli	Bacteria		XL1-blue		-	Agilent
Escherichia coli	Bacteria		BL21		-	Invitrogen
Escherichia coli	Bacteria		SoluBL21		-	Genlantis
