# Supplementary figures and images for: Non-canonical two-step biosynthesis of anti-oomycete indole alkaloids in Kickxellales
Source: Fungal Biol Biotechnol. 2023 Sep 5;10:19. doi: 10.1186/s40694-023-00166-x (PMC10478498; doi:10.1186/s40694-023-00166-x)

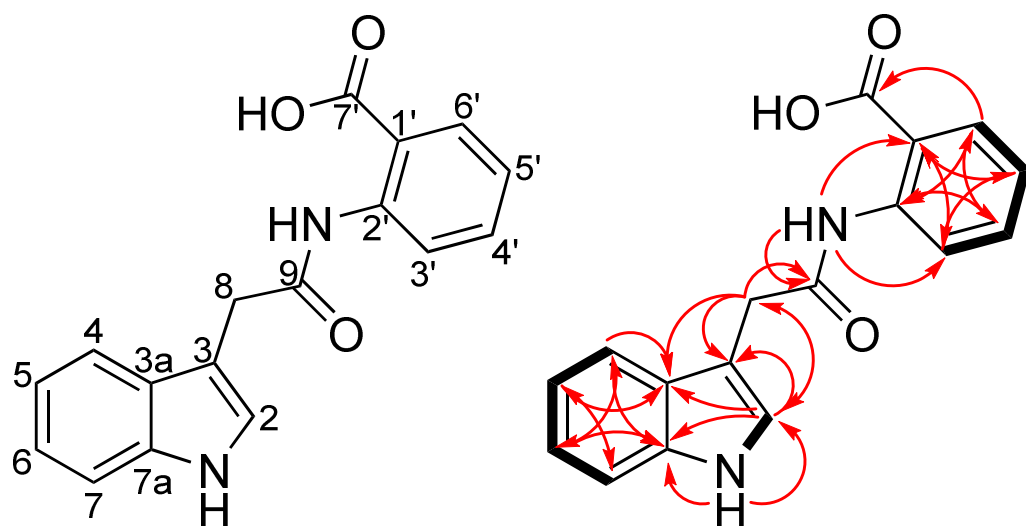

Figure S1. Atom numbering and selected COSY (bold lines) and HMBC (red arrows) in 4.

Supplement: Supplementary file 2 — Additional file 2: Figure S1. Atom numbering and selected COSY (bold lines) and HMBC (red arrows) in 4. [file 40694_2023_166_MOESM2_ESM.pdf]

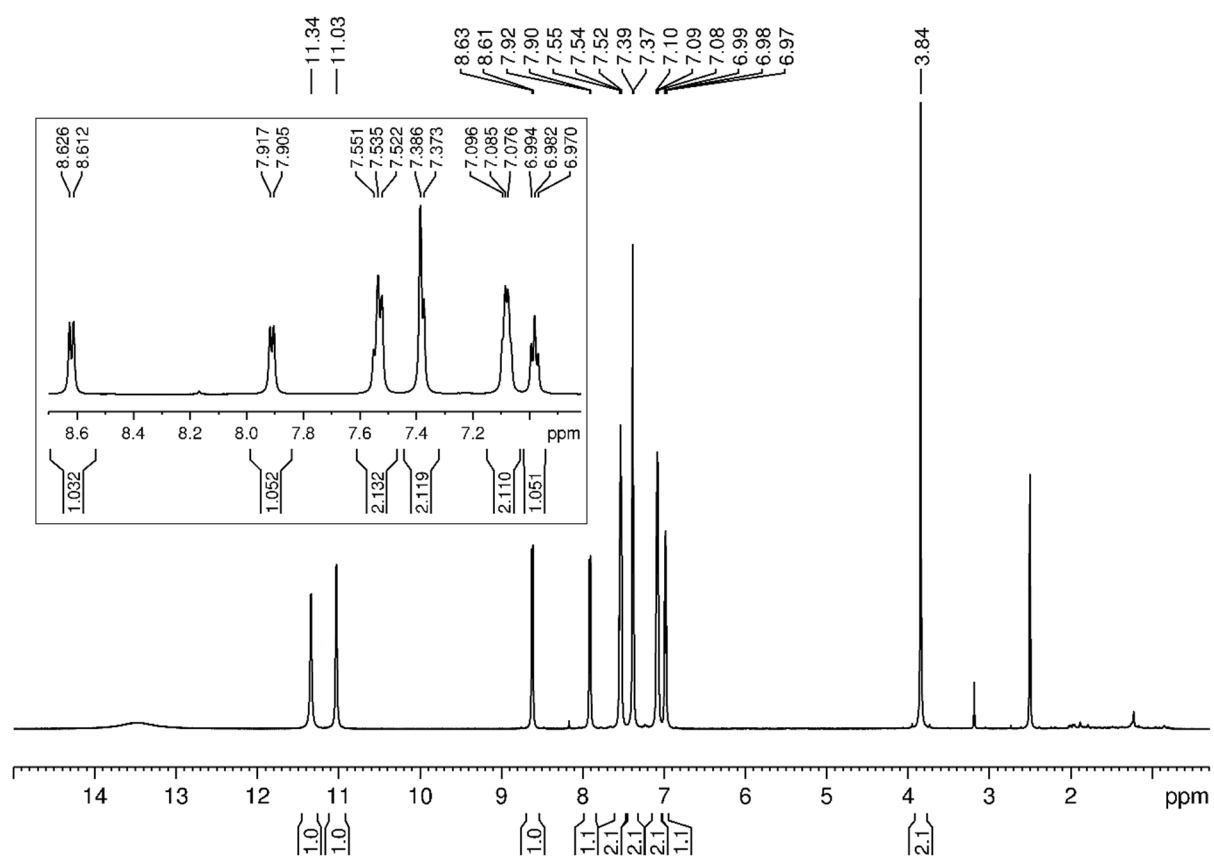

Figure S2.  $^1\text{H}$  NMR spectrum of **4** in  $\text{DMSO-}d_6$ .

Supplement: Supplementary file 3 — Additional file 3: Figure S2. 1H NMR spectrum of 4 in DMSO-d6. [file 40694_2023_166_MOESM3_ESM.pdf]

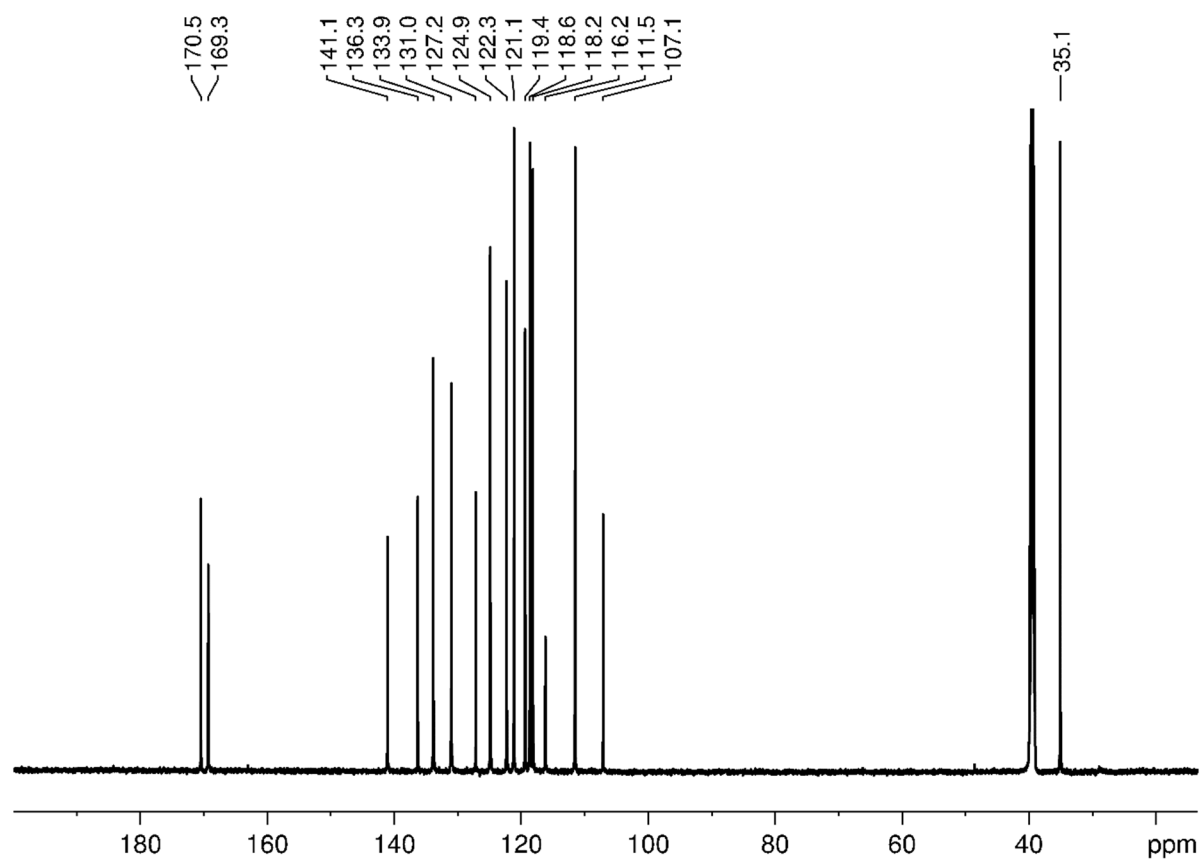

Figure S3. <sup>13</sup>C NMR spectrum of 4 in DMSO-*d*<sub>6</sub>.

Supplement: Supplementary file 4 — Additional file 4: Figure S3. 13C NMR spectrum of 4 in DMSO-d6. [file 40694_2023_166_MOESM4_ESM.pdf]

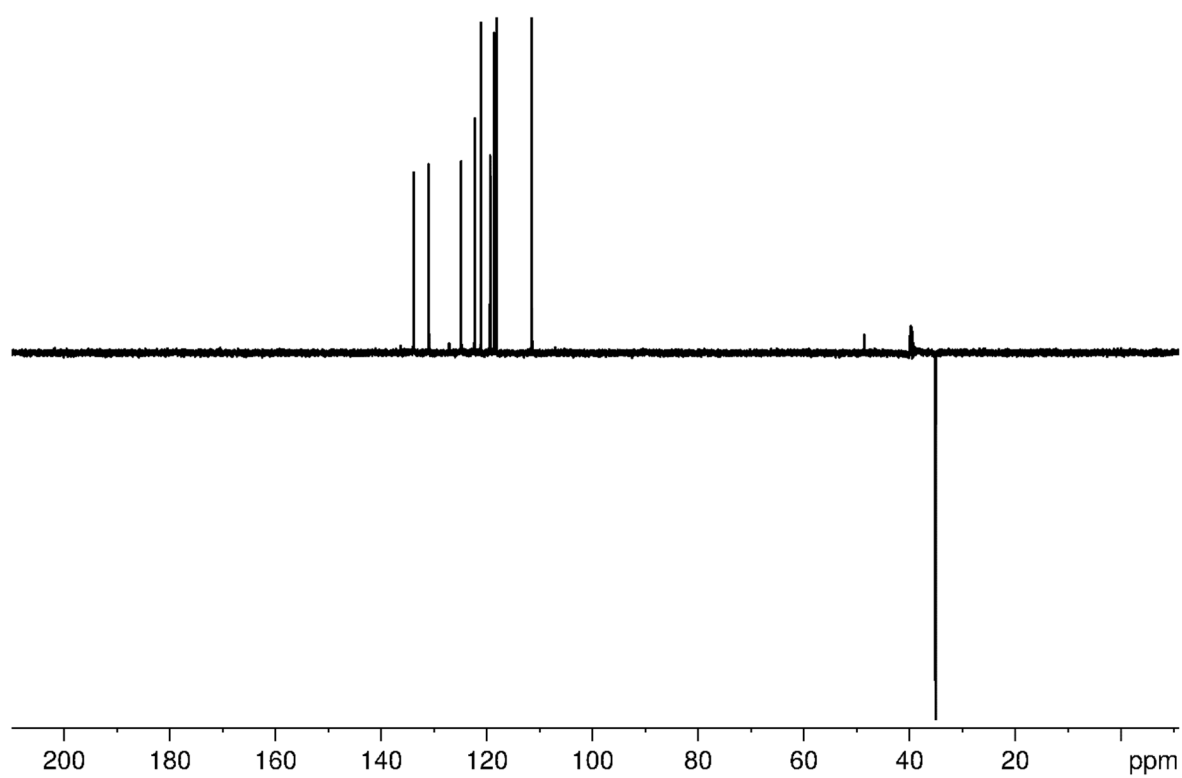

**Figure S4.** DEPT-135 NMR spectrum of **4** in DMSO- $d_6$ .

Supplement: Supplementary file 5 — Additional file 5: Figure S4. DEPT-135 NMR spectrum of 4 in DMSO-d6. [file 40694_2023_166_MOESM5_ESM.pdf]

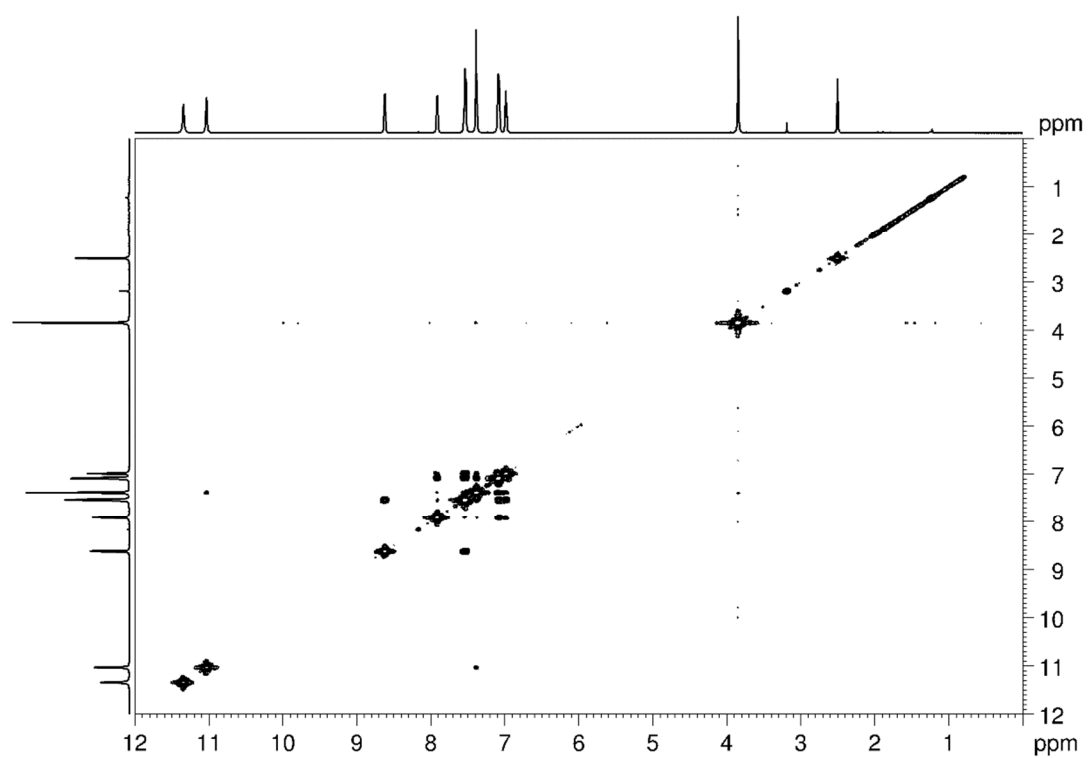

Figure S5.  $^1\text{H}$ - $^1\text{H}$  COSY spectrum of **4** in  $\text{DMSO}-d_6$ .

Supplement: Supplementary file 6 — Additional file 6: Figure S5. 1H-1H COSY spectrum of 4 in DMSO-d6. [file 40694_2023_166_MOESM6_ESM.pdf]

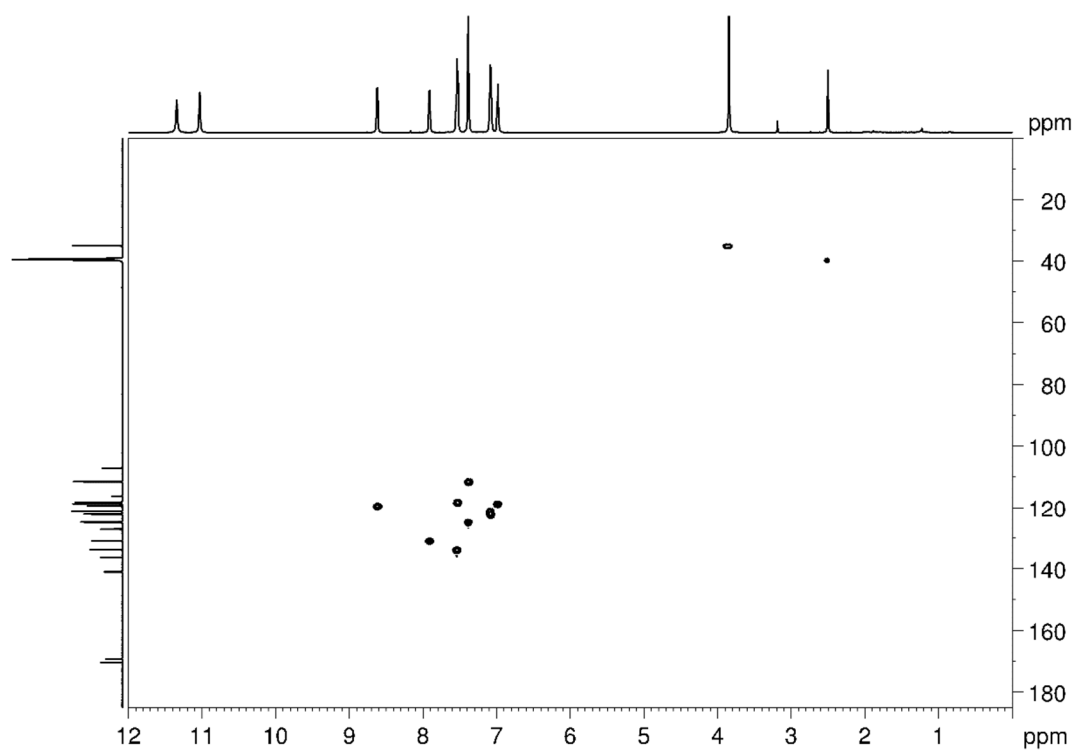

Figure S6.  $^1\text{H}$ - $^{13}\text{C}$  HSQC spectrum of **4** in  $\text{DMSO-}d_6$ .

Supplement: Supplementary file 7 — Additional file 7: Figure S6. 1H-13C HSQC spectrum of 4 in DMSO-d6. [file 40694_2023_166_MOESM7_ESM.pdf]

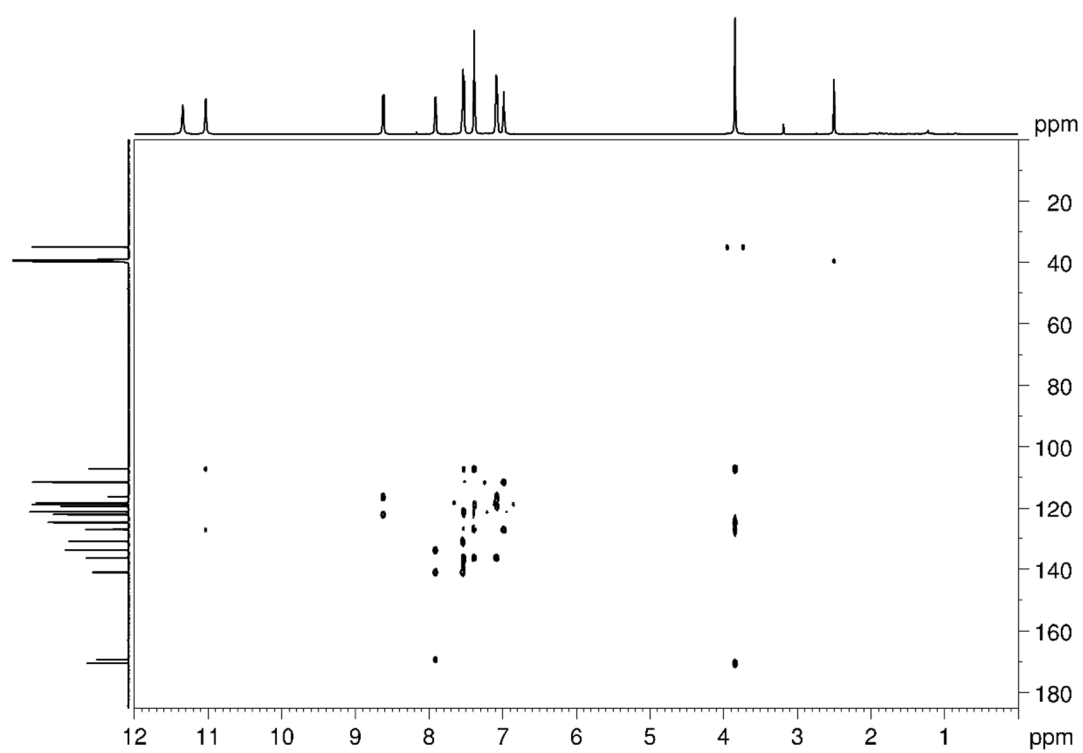

Figure S7.  $^1\text{H}$ - $^{13}\text{C}$  HMBC spectrum of 4 in  $\text{DMSO}-d_6$ .

Supplement: Supplementary file 8 — Additional file 8: Figure S7. 1H-13C HMBC spectrum of 4 in DMSO-d6. [file 40694_2023_166_MOESM8_ESM.pdf]

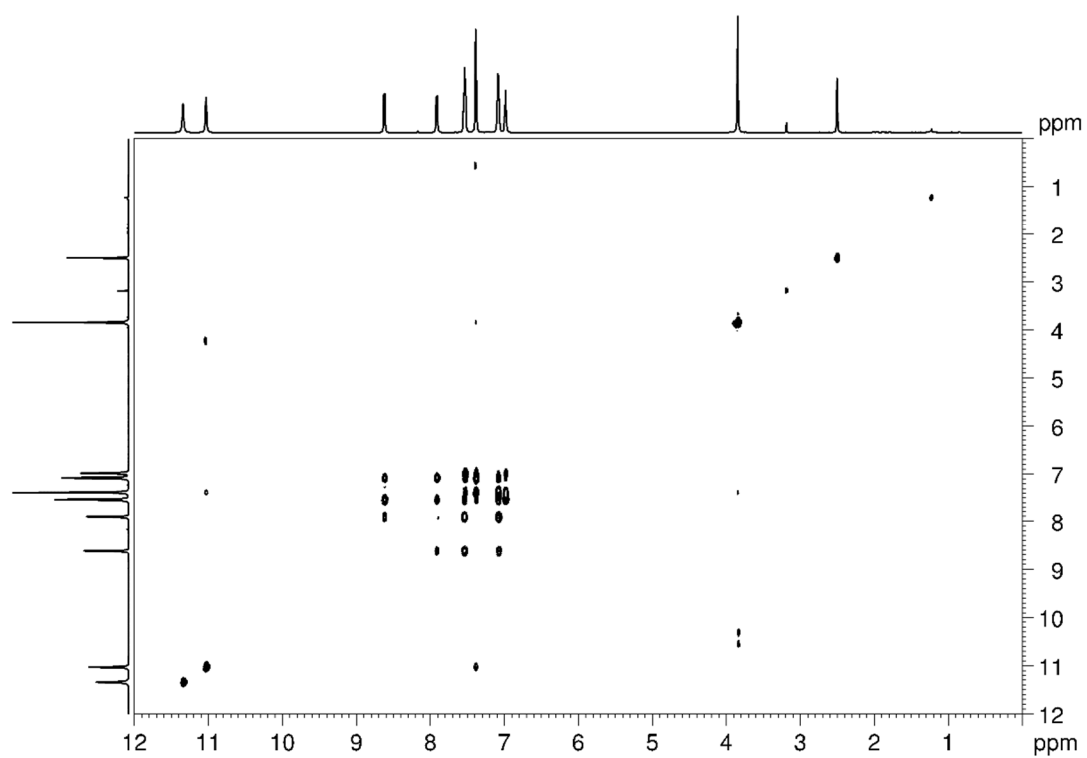

**Figure S8.**  $^1\text{H}$ - $^1\text{H}$  TOCSY spectrum of 4 in  $\text{DMSO}-d_6$ .

Supplement: Supplementary file 9 — Additional file 9: Figure S8. 1H-1H TOCSY spectrum of 4 in DMSO-d6. [file 40694_2023_166_MOESM9_ESM.pdf]

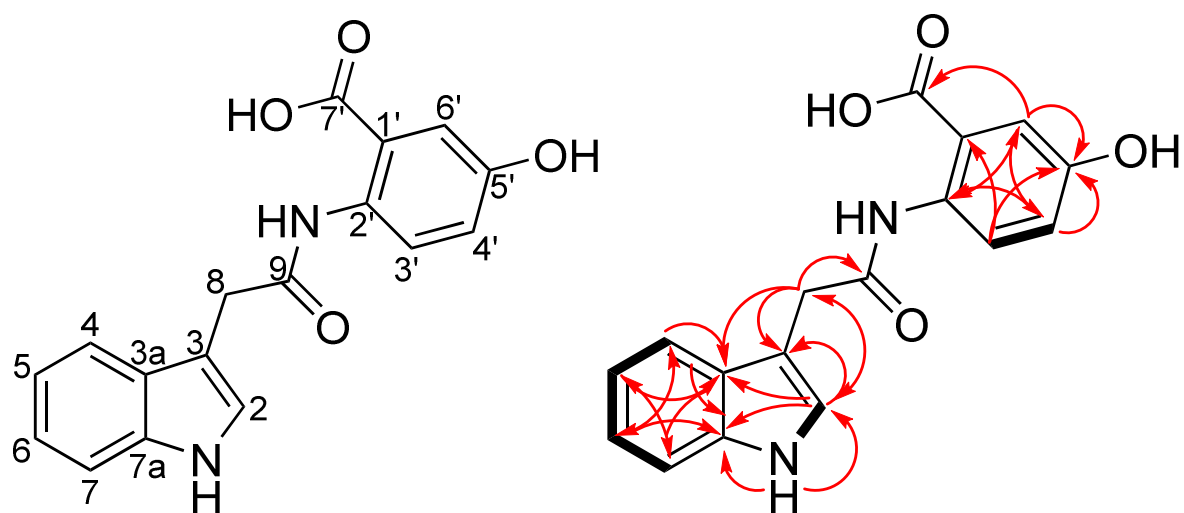

Figure S10. Atom numbering and selected COSY (bold lines) and HMBC (red arrows) in 5.

Supplement: Supplementary file 12 — Additional file 12: Figure S10. Atom numbering and selected COSY (bold lines) and HMBC (red arrows) in 5. [file 40694_2023_166_MOESM12_ESM.pdf]

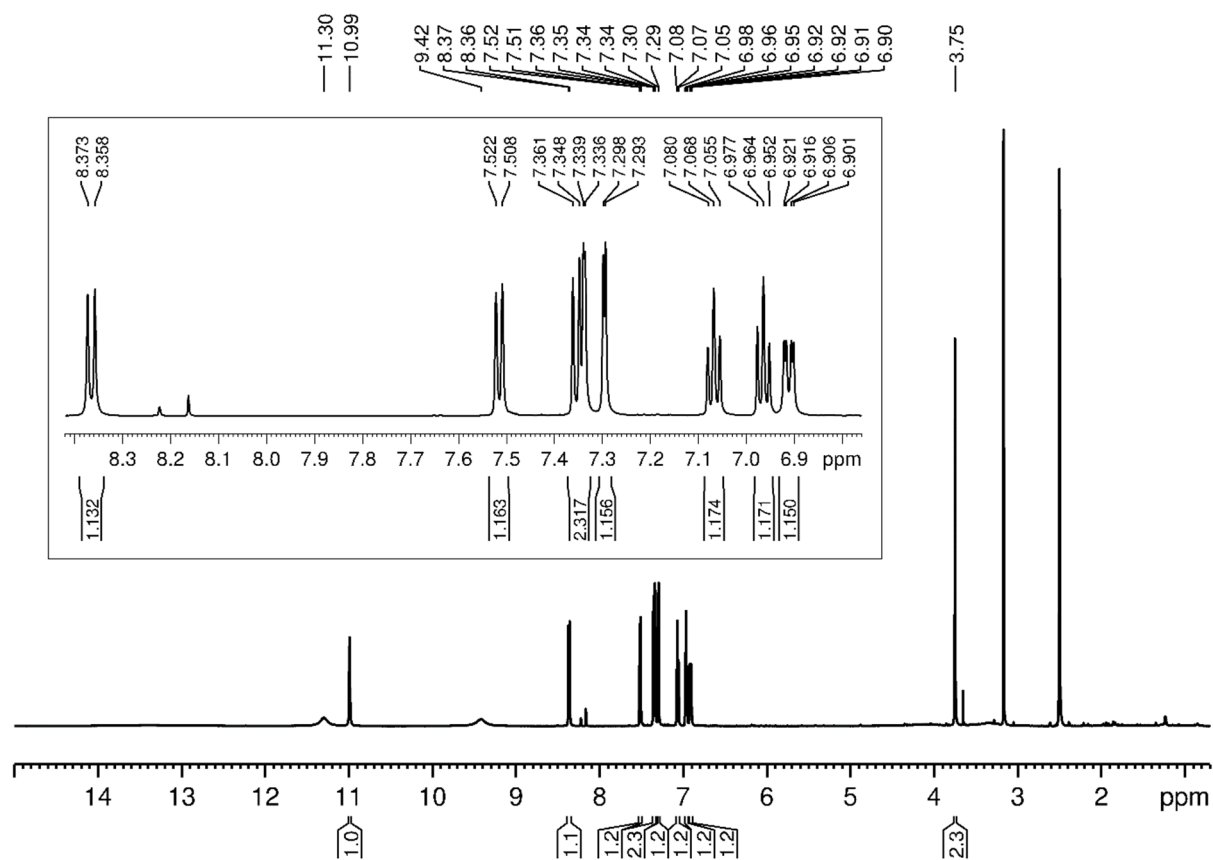

Figure S11.  $^1\text{H}$  NMR spectrum of **5** in  $\text{DMSO-}d_6$ .

Supplement: Supplementary file 13 — Additional file 13: Figure S11. 1H NMR spectrum of 5 in DMSO-d6. [file 40694_2023_166_MOESM13_ESM.pdf]

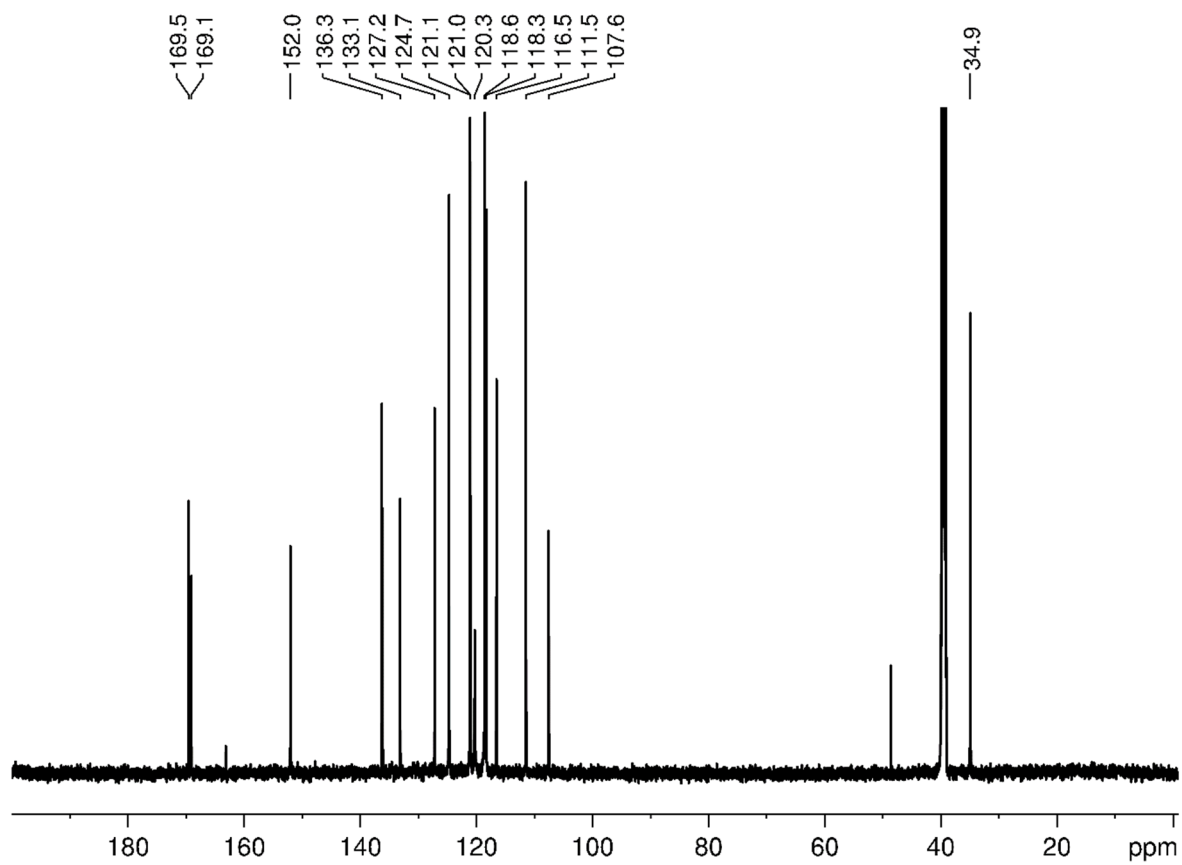

Figure S12. <sup>13</sup>C NMR spectrum of 5 in DMSO-*d*<sub>6</sub>.

Supplement: Supplementary file 14 — Additional file 14: Figure S12. 13C NMR spectrum of 5 in DMSO-d6. [file 40694_2023_166_MOESM14_ESM.pdf]

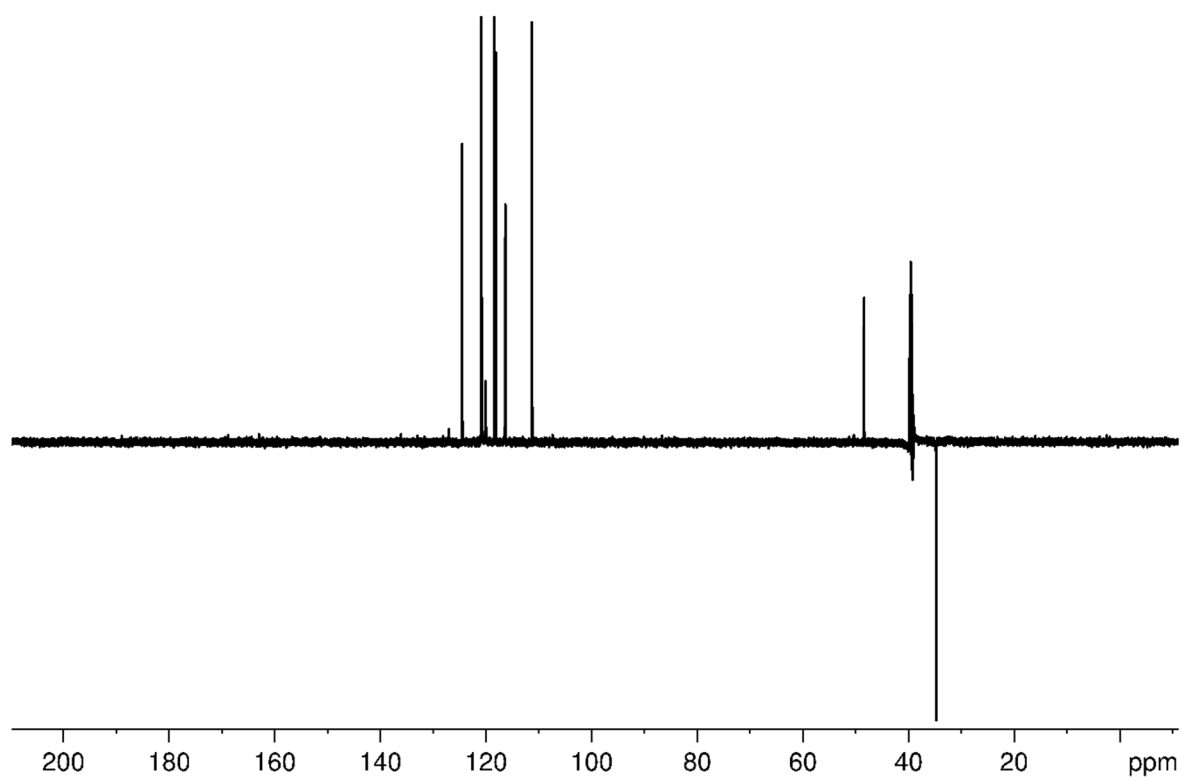

**Figure S13.** DEPT-135 NMR spectrum of **5** in DMSO-*d*<sub>6</sub>.

Supplement: Supplementary file 15 — Additional file 15: Figure S13. DEPT-135 NMR spectrum of 5 in DMSO-d6. [file 40694_2023_166_MOESM15_ESM.pdf]

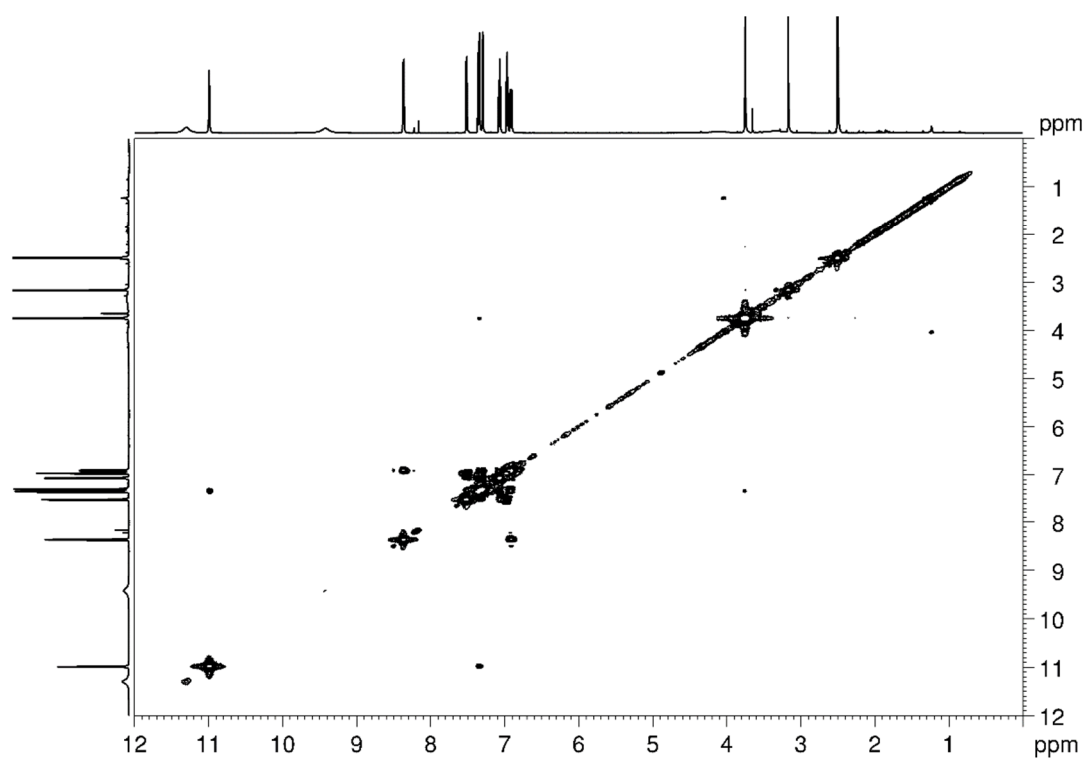

**Figure S14.**  $^1\text{H}$ - $^1\text{H}$  COSY spectrum of 5 in  $\text{DMSO-}d_6$ .

Supplement: Supplementary file 16 — Additional file 16: Figure S14. 1H-1H COSY spectrum of 5 in DMSO-d6. [file 40694_2023_166_MOESM16_ESM.pdf]

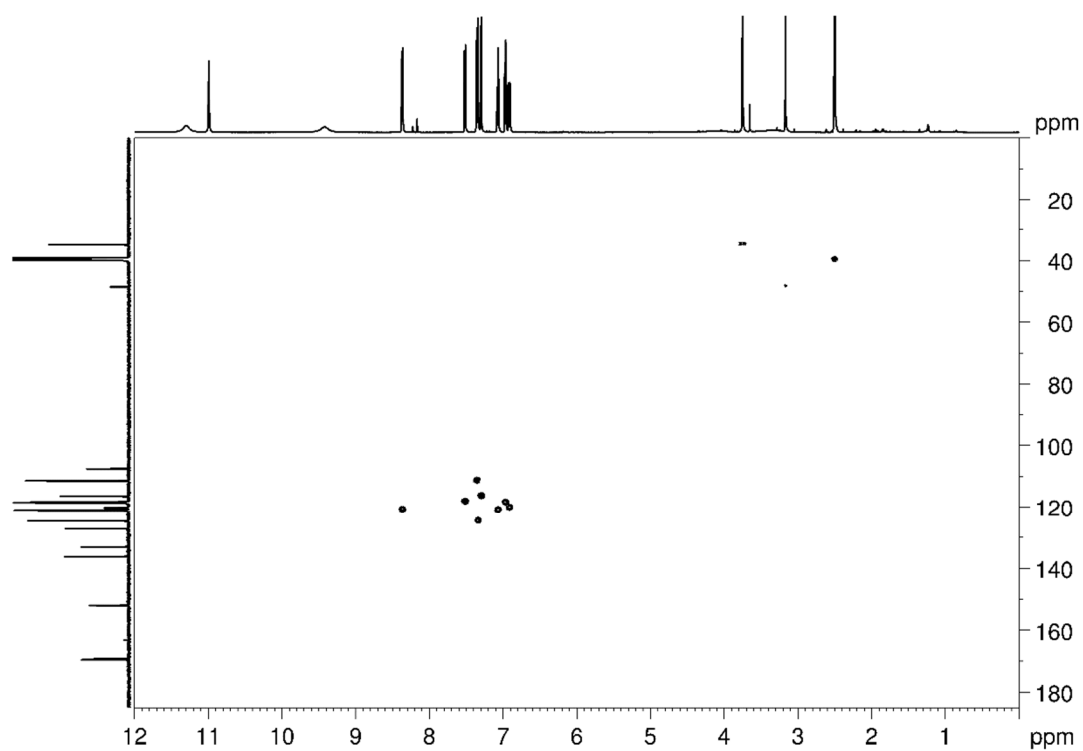

Figure S15.  $^1\text{H}$ - $^{13}\text{C}$  HSQC spectrum of 5 in  $\text{DMSO}-d_6$ .

Supplement: Supplementary file 17 — Additional file 17: Figure S15. 1H-13C HSQC spectrum of 5 in DMSO-d6. [file 40694_2023_166_MOESM17_ESM.pdf]

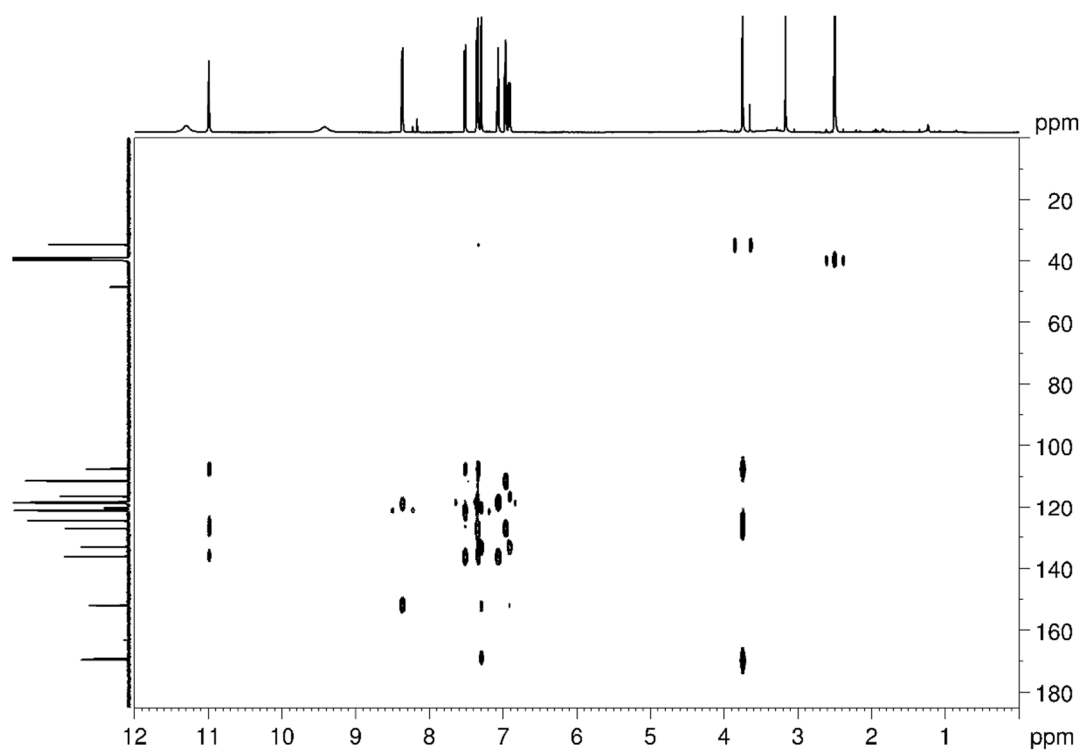

**Figure S16.**  $^1\text{H}$ - $^{13}\text{C}$  HMBC spectrum of 5 in  $\text{DMSO}-d_6$ .

Supplement: Supplementary file 18 — Additional file 18: Figure S16. 1H-13C HMBC spectrum of 5 in DMSO-d6. [file 40694_2023_166_MOESM18_ESM.pdf]

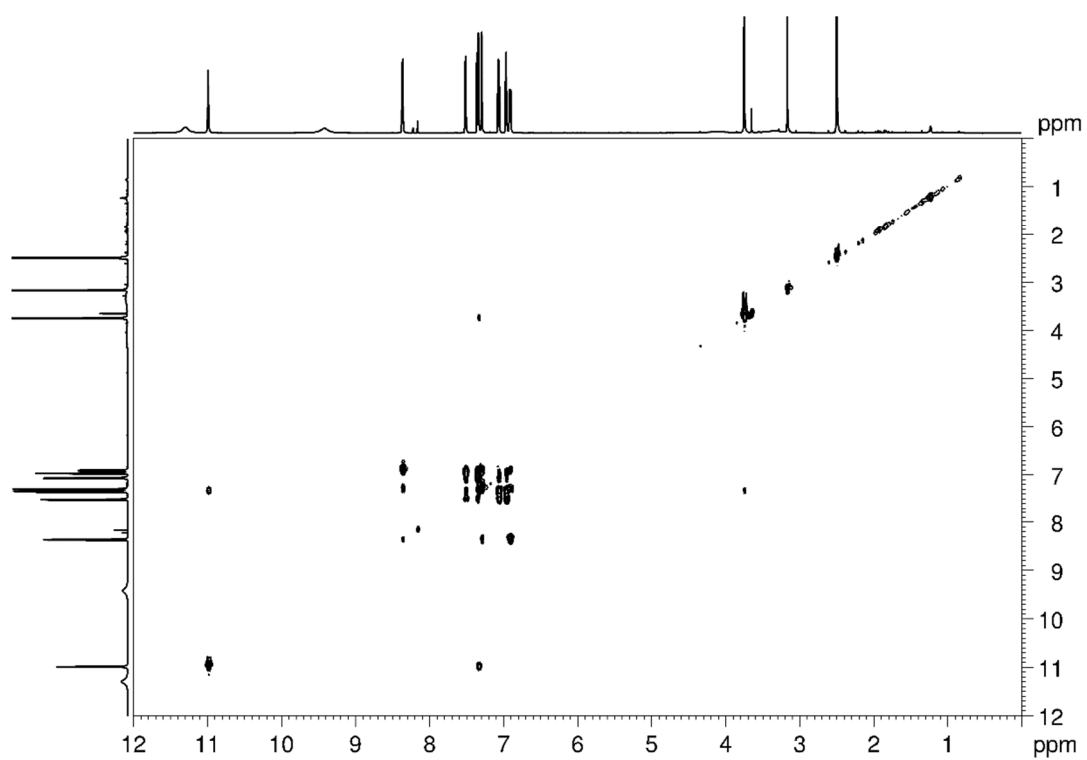

**Figure S17.**  $^1\text{H}$ - $^1\text{H}$  TOCSY spectrum of **5** in  $\text{DMSO-}d_6$

Supplement: Supplementary file 19 — Additional file 19: Figure S17. 1H-1H TOCSY spectrum of 5 in DMSO-d6. [file 40694_2023_166_MOESM19_ESM.pdf]
