# Supplementary material for: Non-canonical two-step biosynthesis of anti-oomycete indole alkaloids in Kickxellales
Source: Fungal Biol Biotechnol. 2023 Sep 5;10:19. doi: 10.1186/s40694-023-00166-x (PMC10478498; doi:10.1186/s40694-023-00166-x)
Supplement: Supplementary file 10 — Additional file 10: Figure S9. ESI-MS/MS spectrum of 4. [file 40694_2023_166_MOESM10_ESM.pdf]

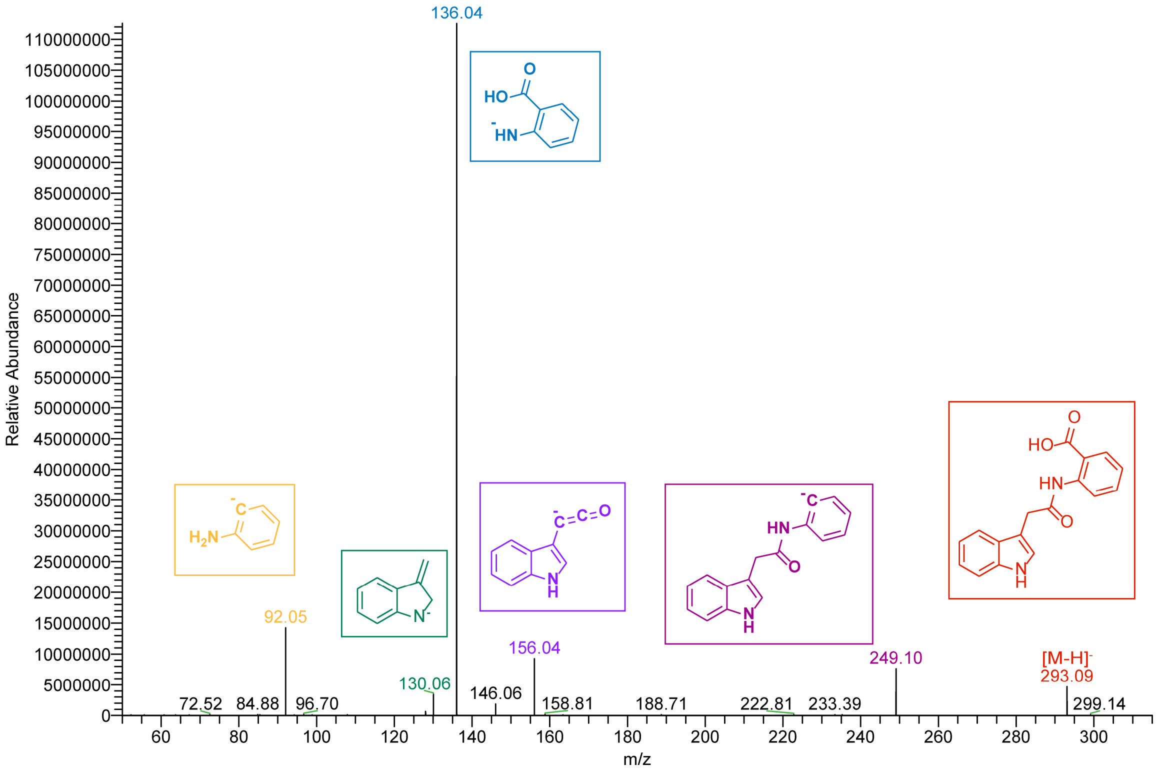

Figure S9. ESI-MS/MS spectrum of 4. Fragmentation was carried out at a higher-energy collisional dissociation (HCD) energy of 30 %.
