# Supplementary material for: Non-canonical two-step biosynthesis of anti-oomycete indole alkaloids in Kickxellales
Source: Fungal Biol Biotechnol. 2023 Sep 5;10:19. doi: 10.1186/s40694-023-00166-x (PMC10478498; doi:10.1186/s40694-023-00166-x)
Supplement: Supplementary file 11 — Additional file 11: Table S2. NMR data of 4 in DMSO-d6. [file 40694_2023_166_MOESM11_ESM.pdf]

Table S2. NMR data of 4 in DMSO-d_6. ^{1}H and ^{13}C NMR spectra were recorded at 600 MHz and 150 MHz, respectively.

	δ ^{13}C [ppm]	δ ^{1}H [ppm], M (*J* [Hz])	COSY	HMBC
indole-NH		11.03, s	2	2, 3, 3a, 7a
2	124.89	7.39, s	indole-NH	3, 3a, 7a, 8
3	107.10			
3a	127.16			
4	118.18	7.52, d (8.1)	5	3a, 6, 7, 7a
5	118.64	6.98, t (7.3)	4, 6	3a, 7
6	121.13	7.09, t (6.9)	5, 7	4, 7a
7	111.51	7.38, d (7.8)	6	5
7a	136.31			
8	35.11	3.84, s		2, 3, 3a, 9
9	170.46			
amide-NH		11.34, s		2‘, 3‘, 8
1‘	116.19			
2‘	141.05			
3‘	119.38	8.62, d (8.3)	4‘	1‘, 2‘, 5‘, 7‘
4‘	133.85	7.54, m	3‘, 5‘	2‘, 6‘
5‘	122.30	7.07, t (6.9)	4‘, 6‘	1‘, 3‘
6‘	131.01	7.91, d (7.5)	5‘	2‘, 4‘, 7‘
7‘	169.29			
