# Supplementary material for: Non-canonical two-step biosynthesis of anti-oomycete indole alkaloids in Kickxellales
Source: Fungal Biol Biotechnol. 2023 Sep 5;10:19. doi: 10.1186/s40694-023-00166-x (PMC10478498; doi:10.1186/s40694-023-00166-x)
Supplement: Supplementary file 20 — Additional file 20: Figure S18. ESI-MS/MS spectrum of 5. [file 40694_2023_166_MOESM20_ESM.pdf]

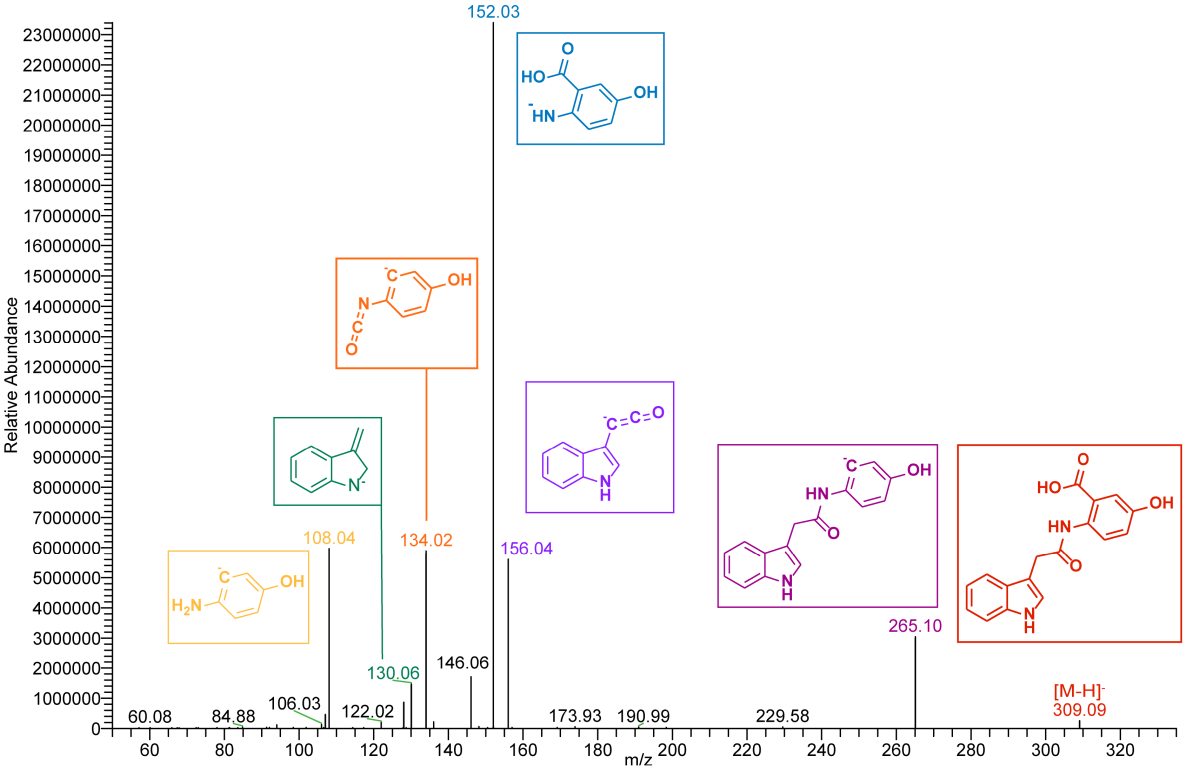

Figure S18. ESI-MS/MS spectrum of 5. Fragmentation was carried out at a higher-energy collisional dissociation (HCD) energy of 35 %.
