# Supplementary material for: Non-canonical two-step biosynthesis of anti-oomycete indole alkaloids in Kickxellales
Source: Fungal Biol Biotechnol. 2023 Sep 5;10:19. doi: 10.1186/s40694-023-00166-x (PMC10478498; doi:10.1186/s40694-023-00166-x)
Supplement: Supplementary file 21 — Additional file 21: Table S3. NMR data of 5 in DMSO-d6. [file 40694_2023_166_MOESM21_ESM.pdf]

Table S3. NMR data of 5 in DMSO-*d*$_6$. ^{1}H and ^{13}C NMR spectra were recorded at 600 MHz and 150 MHz, respectively. br = broad signal. * = tentative, overlapping signal.

	δ ^{13}C [ppm]	δ ^{1}H [ppm], M (*J* [Hz])	COSY	HMBC
indole-NH		10.99, s	2	2, 3, 3a, 7a
2	124.72	7.34, d (1.9)	indole-NH	3, 3a, 7a, 8
3	107.56			
3a	127.20			
4	118.30	7.52, d (7.9)	5	3, 6, 7a
5	*118.58	6.96, t (7.4)	4, 6	3a, 7
6	121.09	7.07, t (7.5)	5, 7	4, 7a
7	111.48	7.35, d (8.2)	6	3a, 5
7a	136.27			
8	34.94	3.75, s		2, 3, 3a, 9
9	169.53			
amide-NH		11.30, s (br)		
1ʻ	*118.58			
2ʻ	133.15			
3ʻ	121.01	8.37, d (9.0)	4ʻ	1ʻ, 5ʻ, 7ʻ
4ʻ	120.27	6.91, dd (9.0, 2.9)	3ʻ	2ʻ, 5ʻ, 6ʻ
5ʻ	152.01			
OH		9.42, s (br)		
6ʻ	116.53	7.30, d (2.9 Hz)		2ʻ, 4ʻ, 5ʻ, 7ʻ
7ʻ	169.13			
