# Supplementary material for: Non-canonical two-step biosynthesis of anti-oomycete indole alkaloids in Kickxellales
Source: Fungal Biol Biotechnol. 2023 Sep 5;10:19. doi: 10.1186/s40694-023-00166-x (PMC10478498; doi:10.1186/s40694-023-00166-x)
Supplement: Supplementary file 22 — Additional file 22: Figure S19. GC-MS/MS spectrum of ILA (1). [file 40694_2023_166_MOESM22_ESM.pdf]

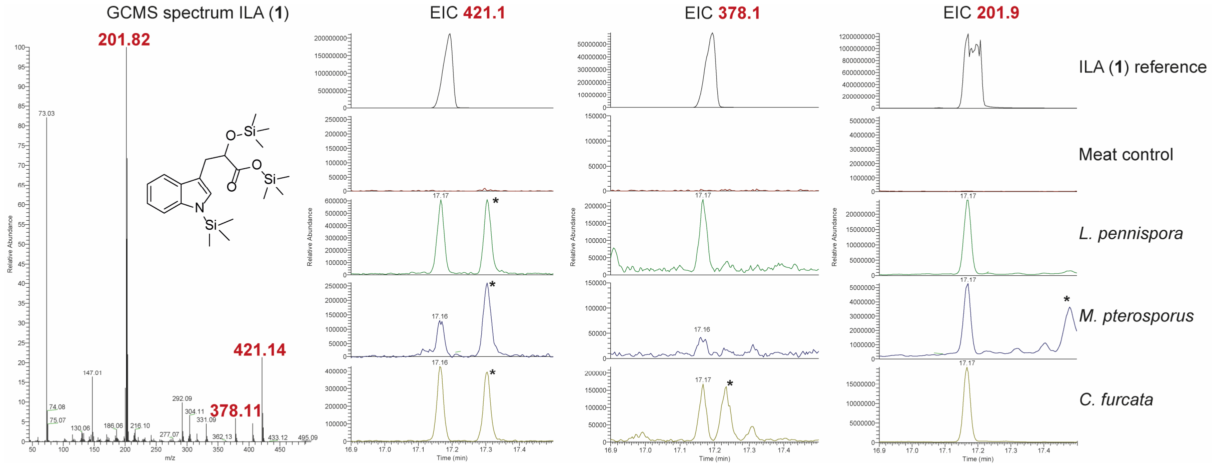

Figure S19. GC-MS/MS spectrum of ILA (1). 1 from *Linderina pennispora, Martensiomyces pterosporus* and *Coemansia furcata* were silylated with *N*-Methyl-*N*-(trimethylsilyl)trifluoroacetamide (MSTFA) prior to GC analysis. The parent MS fragment and the daughter fragments were verified against an analogously silylated commercial ILA standard. An extract of non-inoculated meat medium served as negative control. Asterisks indicate equi-ionic, but irrelevant peaks.
