# Supplementary material for: Non-canonical two-step biosynthesis of anti-oomycete indole alkaloids in Kickxellales
Source: Fungal Biol Biotechnol. 2023 Sep 5;10:19. doi: 10.1186/s40694-023-00166-x (PMC10478498; doi:10.1186/s40694-023-00166-x)
Supplement: Supplementary file 24 — Additional file 24: Figure S21. GC-MS/MS spectrum of IAA (3). [file 40694_2023_166_MOESM24_ESM.pdf]

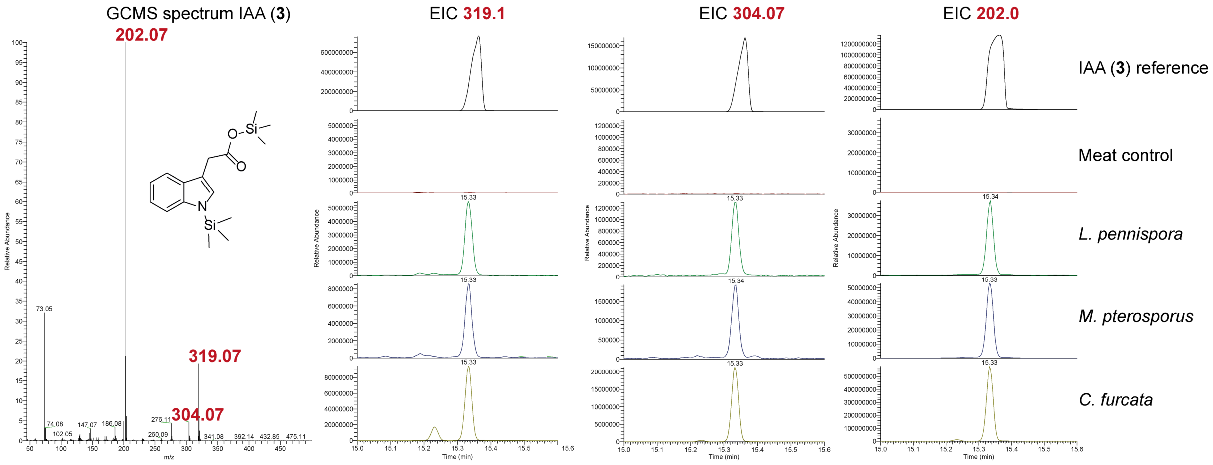

Figure S21. GC-MS/MS spectrum of IAA (3). 3 from *Linderina pennispora, Martensiomyces pteropsorus* and *Coemansia furcata* were silylated with *N*-Methyl-*N*-(trimethylsilyl)trifluoroacetamide (MSTFA) prior to GC analysis. The parent MS fragment and the daughter fragments were verified against an analogously silylated commercial IAA standard. An extract of non-inoculated meat medium served as negative control.
