# Supplementary material for: Non-canonical two-step biosynthesis of anti-oomycete indole alkaloids in Kickxellales
Source: Fungal Biol Biotechnol. 2023 Sep 5;10:19. doi: 10.1186/s40694-023-00166-x (PMC10478498; doi:10.1186/s40694-023-00166-x)
Supplement: Supplementary file 25 — Additional file 25: Figure S22. Production of lindolin A and B in Kickxellales and related species. [file 40694_2023_166_MOESM25_ESM.pdf]

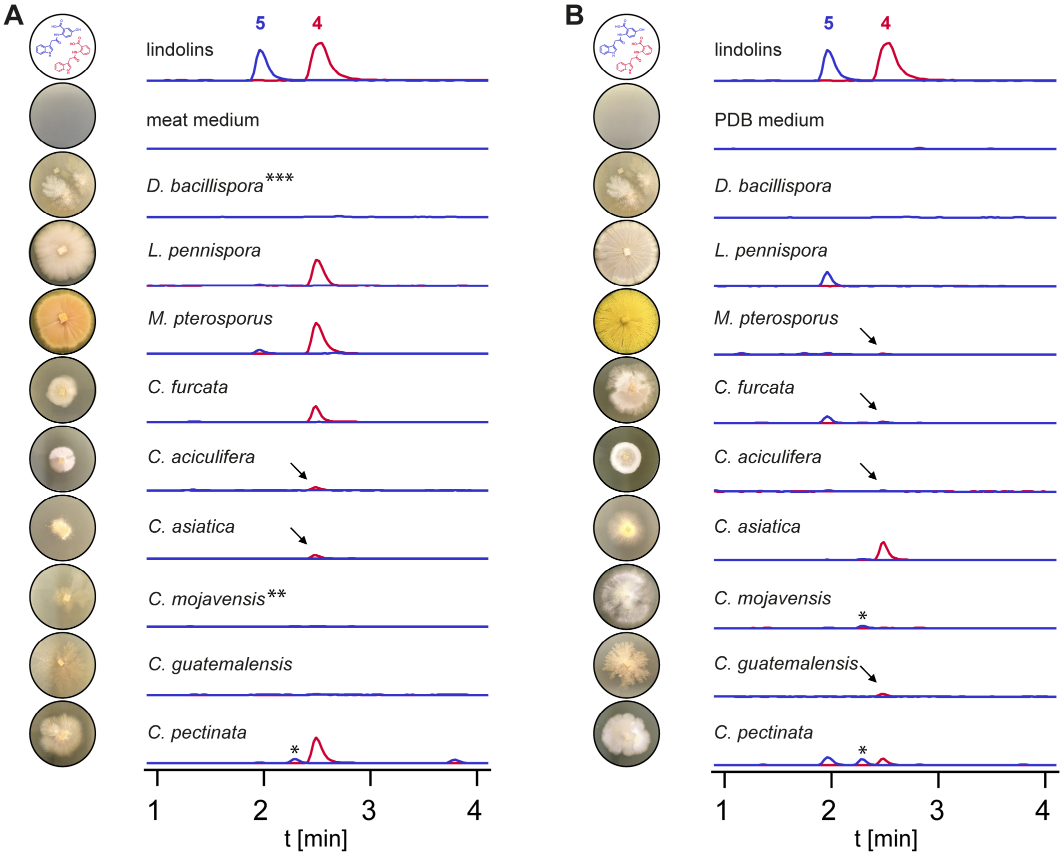

Figure S22. Production of lindolin A and B in Kickxellales and related species. Kickxellales (*Linderina pennispora, Martensiomyces pterosporus, Coemansia furcata, Coemansia aciculifera, Coemansia asiatica, Coemansia mojavensis, Coemansia guatemalensis, Coemansia pectinata*) were submersely grown in meat medium (**A**) or PDB medium (**B**) under shaking conditions and metabolite crude extracts of culture supernatants were chromatographed. Pictures of the corresponding agar cultures are shown. Chromatograms were recorded by UHPLC-MS and overlaid extracted ion chromatograms were shown for *m/z* 293 $[M\text{–H}]^-$ and *m/z* 309 $[M\text{–H}]^-$ for **4** (red trace) and **5** (blue trace), respectively. Non-inoculated media served as negative controls. * These signals indicate irrelevant peaks with an equivalent mass as **5**. ** *C. mojavensis* does not produce lindolins in shaking culture but on agar plates (not shown). *** The mycoparasite *Dimargaris bacillispora* (order Dimargaritales) was cultivated on its host *Cokeromyces recurvatus* on V8 medium and does not produce lindolins. All chromatograms were normalized against the most intensive signal of **4** in *M. pterosporus*.
