# Supplementary material for: Non-canonical two-step biosynthesis of anti-oomycete indole alkaloids in Kickxellales
Source: Fungal Biol Biotechnol. 2023 Sep 5;10:19. doi: 10.1186/s40694-023-00166-x (PMC10478498; doi:10.1186/s40694-023-00166-x)
Supplement: Supplementary file 26 — Additional file 26: Figure S23. Antimicrobial activities of 4 and 5. [file 40694_2023_166_MOESM26_ESM.pdf]

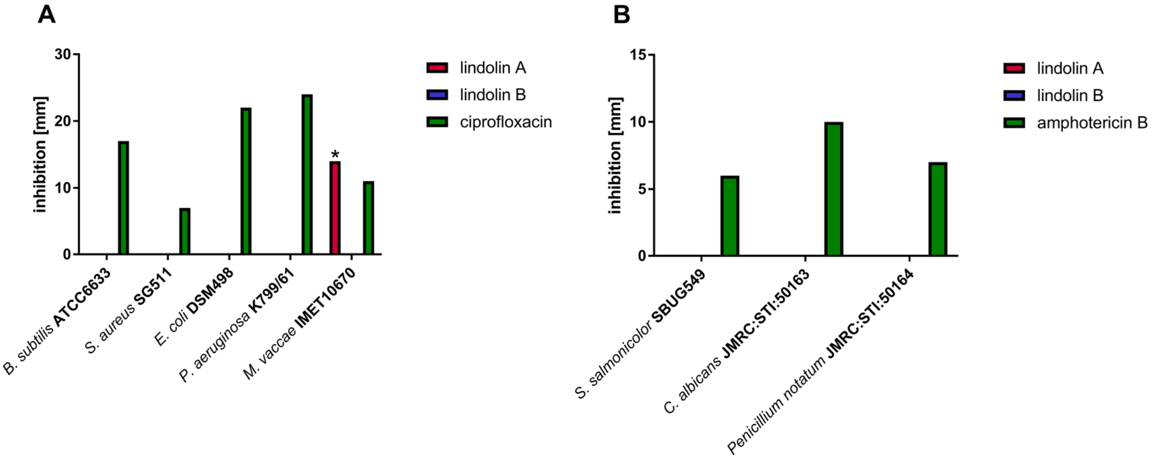

Figure S23. Antimicrobial activities of 4 and 5. Five bacterial (**A**) and three fungal strains (**B**) were tested against **4** and **5** (1000 µg mL^{-1} each). Ciprofloxacin (5 µg mL^{-1}) and amphotericin B (10 µg mL^{-1}) served as controls. * Inhibition zone contained small colonies (only partial inhibition).
