# Supplementary material for: Non-canonical two-step biosynthesis of anti-oomycete indole alkaloids in Kickxellales
Source: Fungal Biol Biotechnol. 2023 Sep 5;10:19. doi: 10.1186/s40694-023-00166-x (PMC10478498; doi:10.1186/s40694-023-00166-x)
Supplement: Supplementary file 27 — Additional file 27: Figure S24. Antiproliferative and cytotoxic activities of 4 and 5 against mammalian cells. [file 40694_2023_166_MOESM27_ESM.pdf]

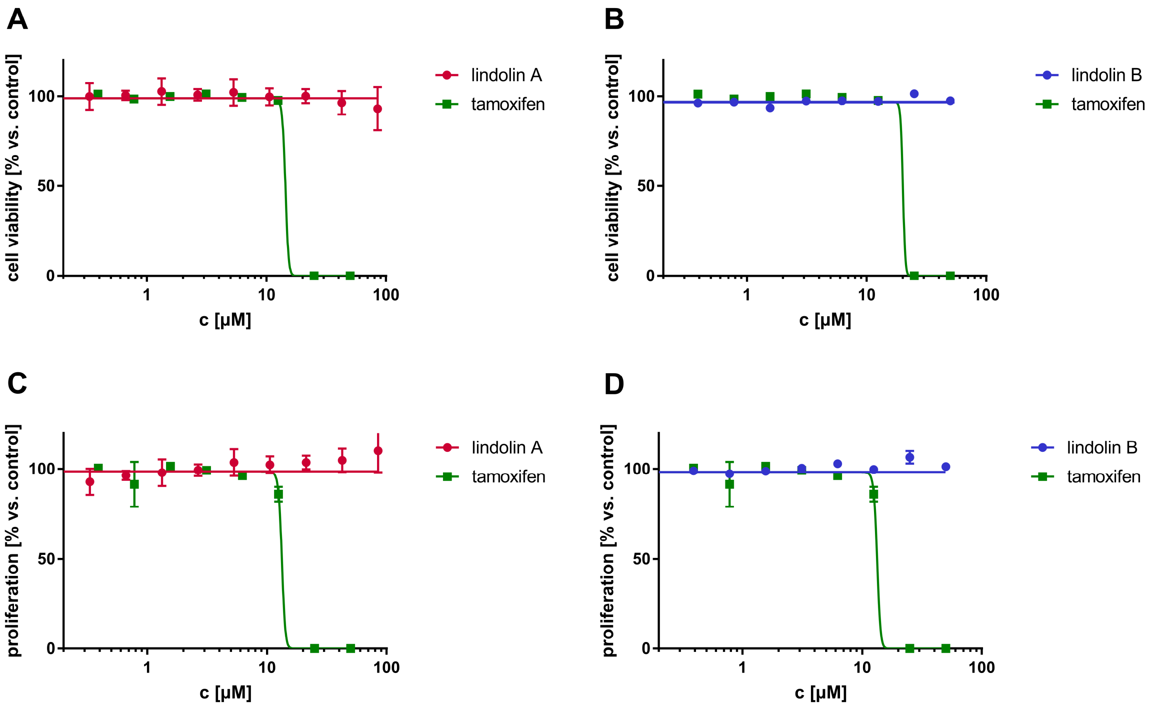

Figure S24. Antiproliferative and cytotoxic activities of 4 and 5 against mammalian cells. HeLa (**A** and **B**) and HUVEC (**C** and **D**) cell lines were tested against binary dilutions of **4** (**A** and **C**) and **5** (**B** and **D**) up to 100 µg mL^{-1}. Tamoxifen (up to 50 µg mL^{-1}) served as control.
