# Supplementary material for: Non-canonical two-step biosynthesis of anti-oomycete indole alkaloids in Kickxellales
Source: Fungal Biol Biotechnol. 2023 Sep 5;10:19. doi: 10.1186/s40694-023-00166-x (PMC10478498; doi:10.1186/s40694-023-00166-x)
Supplement: Supplementary file 28 — Additional file 28: Figure S25. Plant growth activating and IAA-interfering properties of IAA (3), lindolin A (4) and lindolin B (5) on radish seedlings. [file 40694_2023_166_MOESM28_ESM.pdf]

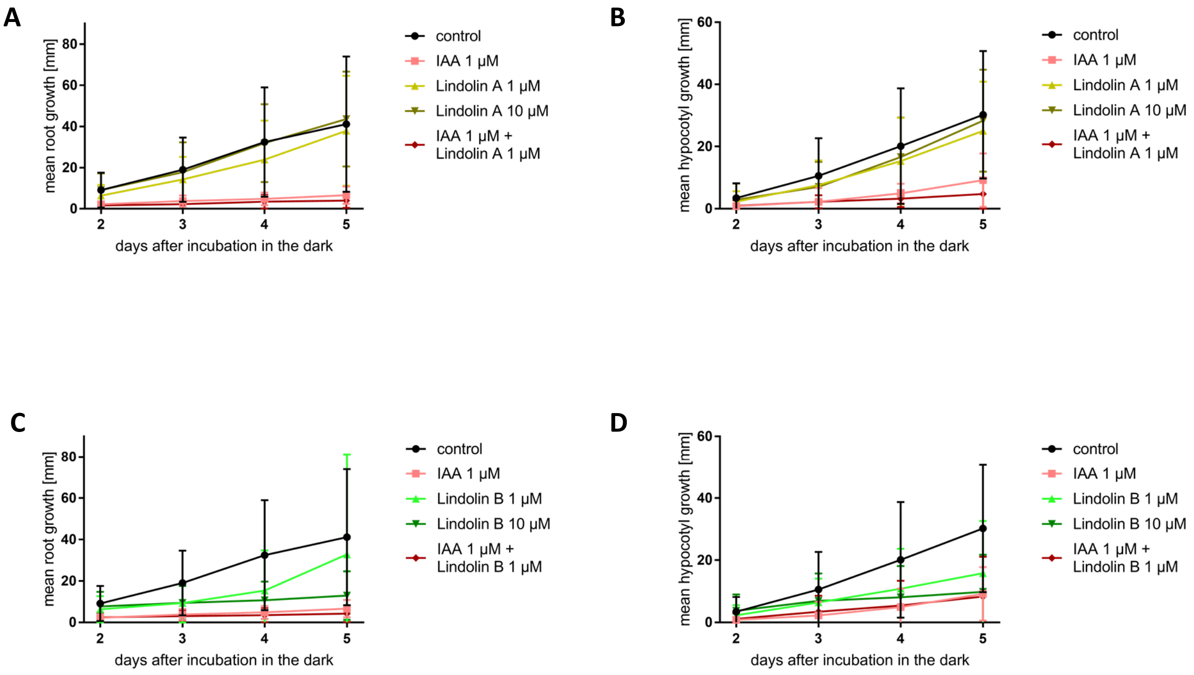

Figure S25. Plant growth activating and IAA-interfering properties of IAA (3), lindolin A (4) and lindolin B (5) on radish seedlings. Radish seedlings were incubated either with **3** (1 µM), **4** (1 and 10 µM) and **3** (1 µM) + **4** (1 µM) (**A** and **B**) or with **3** (1 µM), **5** (1 and 10 µM) and **3** (1 µM) + **5** (1 µM) (**C** and **D**). As positive control, the medium was kept non-supplemented. Root growth (**A** and **C**) and shoot growth (**B** and **D**) was determined over a period of 5 days after cold-shock incubation at 4 °C in the dark. Both **4** and **5** neither affect root or shoot growth significantly nor interfere with the IAA-mediated root and shoot shortening.
