# Supplementary material for: Non-canonical two-step biosynthesis of anti-oomycete indole alkaloids in Kickxellales
Source: Fungal Biol Biotechnol. 2023 Sep 5;10:19. doi: 10.1186/s40694-023-00166-x (PMC10478498; doi:10.1186/s40694-023-00166-x)
Supplement: Supplementary file 29 — Additional file 29: Figure S26. Initial screening for antifungal and anti-oomycete properties of lindolin A (4). [file 40694_2023_166_MOESM29_ESM.pdf]

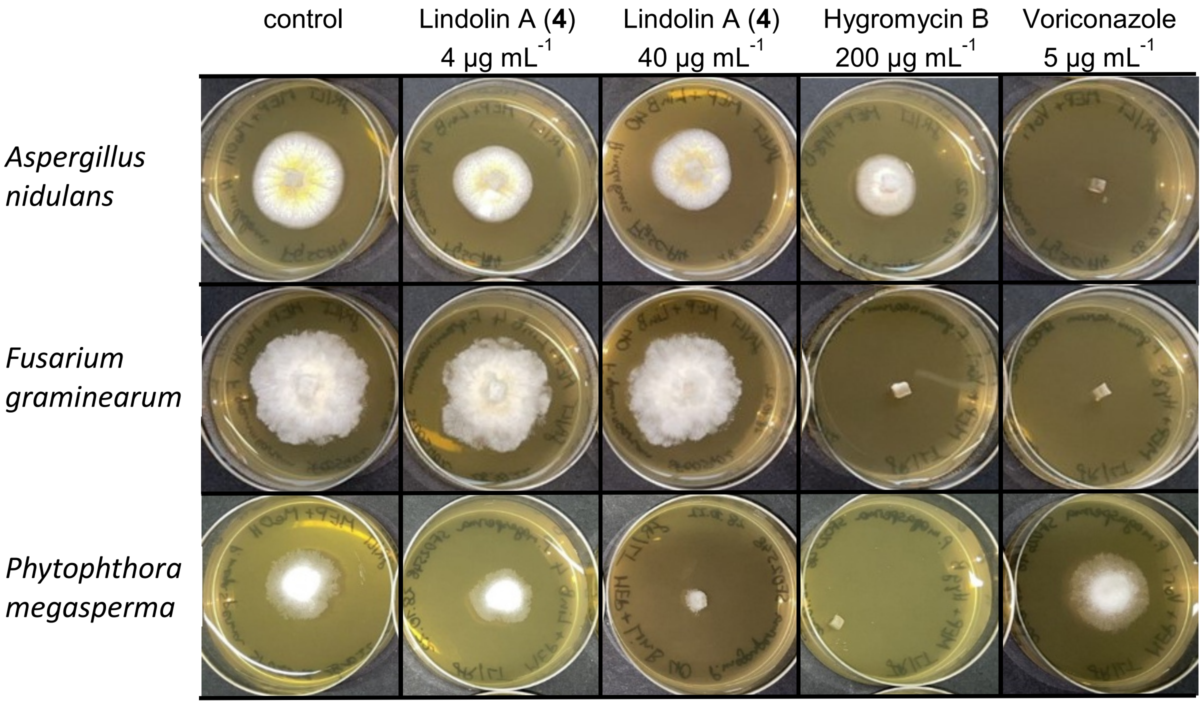

Figure S26. Initial screening for antifungal and anti-oomycete properties of lindolin A (4). The ascomycetes *Aspergillus nidulans* (saprobiont) and *Fusarium graminearum* (phytopathogen) and the oomycete *Phytophthora megasperma* (phytopathogen) were cultivated on MEP medium. The medium was supplemented with 4 or 40 µg mL^{-1} **4**, 200 µg mL^{-1} hygromycin B or 5 µg mL^{-1} voriconazole (negative controls) or was left unmodified (positive control).
