# Supplementary material for: Non-canonical two-step biosynthesis of anti-oomycete indole alkaloids in Kickxellales
Source: Fungal Biol Biotechnol. 2023 Sep 5;10:19. doi: 10.1186/s40694-023-00166-x (PMC10478498; doi:10.1186/s40694-023-00166-x)
Supplement: Supplementary file 30 — Additional file 30: Figure S27. Determination of the minimal inhibitory concentration (MIC50) of lindolin A (4) on oomycetes. [file 40694_2023_166_MOESM30_ESM.pdf]

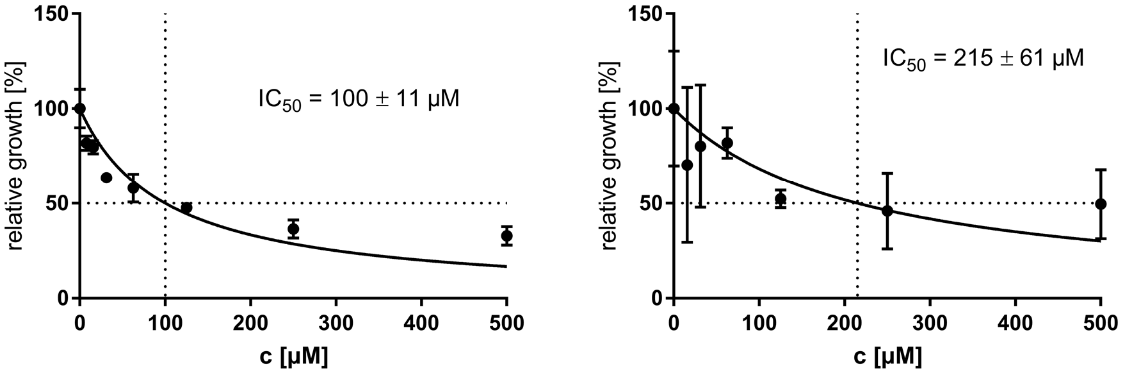

Figure S27. Determination of the minimal inhibitory concentration (MIC_{50}) of Lindolin A (4) on oomycetes. Serial binary dilutions of 7.8 - 500 µM of **4** were tested against *Phytophthora megasperma* (**A**) and *Pythium macrosporum* (**B**) cultivated on PDB agar.
