# Supplementary material for: Non-canonical two-step biosynthesis of anti-oomycete indole alkaloids in Kickxellales
Source: Fungal Biol Biotechnol. 2023 Sep 5;10:19. doi: 10.1186/s40694-023-00166-x (PMC10478498; doi:10.1186/s40694-023-00166-x)
Supplement: Supplementary file 31 — Additional file 31: Table S4. BLAST search for homologous genes required for IAA production in Kickxellales. [file 40694_2023_166_MOESM31_ESM.pdf]

Table S4. BLAST search for homologous genes required for IAA production in Kickxellales.

**Gene query of *Tricholoma vaccinum***			**homolog in *Linderina pennispora***		**homolog in *Martensiomyces pterosporus***		**homolog in *Coemansia furcata***	
name	putative encoded enzyme	accession number	accession number + locus tag	e value	accession number + locus tag	e value	accession number + locus tag	e value
tam1	tryptophan aminotransferase	AJP77090.1	XP_040740547.1 DL89DRAFT_324965	1×10^{-38}	KAI8325817.1 GQ54DRAFT_177899	1×10^{-46}	KAJ2834758.1 GGI24_000220	1×10^{-42}
ipd1	indole-3-pyruvic acid decarboxylase	AJP77091.1	XP_040745548.1 DL89DRAFT_291355	5×10^{-3}	KAI8324123.1 GQ54DRAFT_257134	3×10^{-5}	KAJ2813856.1 H4S07_000370	4×10^{-6}
ald1	aldehyde dehydrogenase	ADY86395.1	XP_040743042.1 DL89DRAFT_174619	4×10^{-114}	KAI8322856.1 GQ54DRAFT_12901	7×10^{-110}	KAJ2812286.1 H4S07_001508	5×10^{-107}
