# Supplementary material for: Non-canonical two-step biosynthesis of anti-oomycete indole alkaloids in Kickxellales
Source: Fungal Biol Biotechnol. 2023 Sep 5;10:19. doi: 10.1186/s40694-023-00166-x (PMC10478498; doi:10.1186/s40694-023-00166-x)
Supplement: Supplementary file 32 — Additional file 32: Figure S28. Quantification of IAA (3), lindolin A (4) and lindolin (5) in cultures of L. pennispora. [file 40694_2023_166_MOESM32_ESM.pdf]

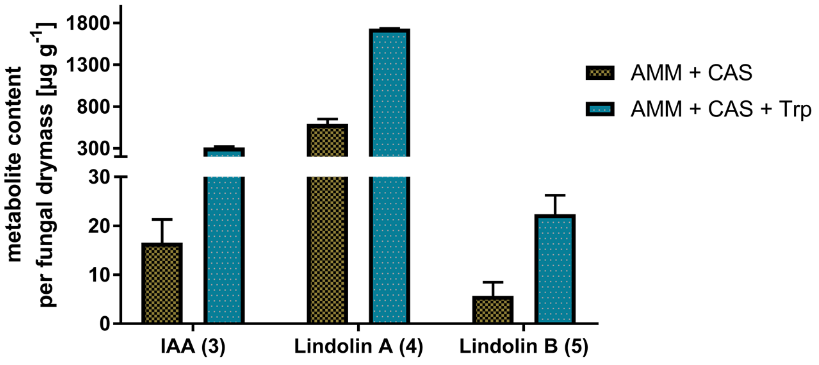

Figure S28. Quantification of IAA (3), lindolin A (4) and lindolin (5) in cultures of *L. pennispora*. The fungus was cultivated for three days on *Aspergillus* Minimal Medium (AMM) with casamino acids (CAS). The medium was optionally supplemented with 5 mM Trp which strikingly enhanced the production of all three metabolites. Note that the titers of **4** can reach up to 0.18 % of the fungal dry biomass.
