# Supplementary material for: Non-canonical two-step biosynthesis of anti-oomycete indole alkaloids in Kickxellales
Source: Fungal Biol Biotechnol. 2023 Sep 5;10:19. doi: 10.1186/s40694-023-00166-x (PMC10478498; doi:10.1186/s40694-023-00166-x)
Supplement: Supplementary file 33 — Additional file 33: Figure S29. SDS polyacrylamid gel electrophoresis (SDS-PAGE) of purified His6-tagged LinA and LinB. [file 40694_2023_166_MOESM33_ESM.pdf]

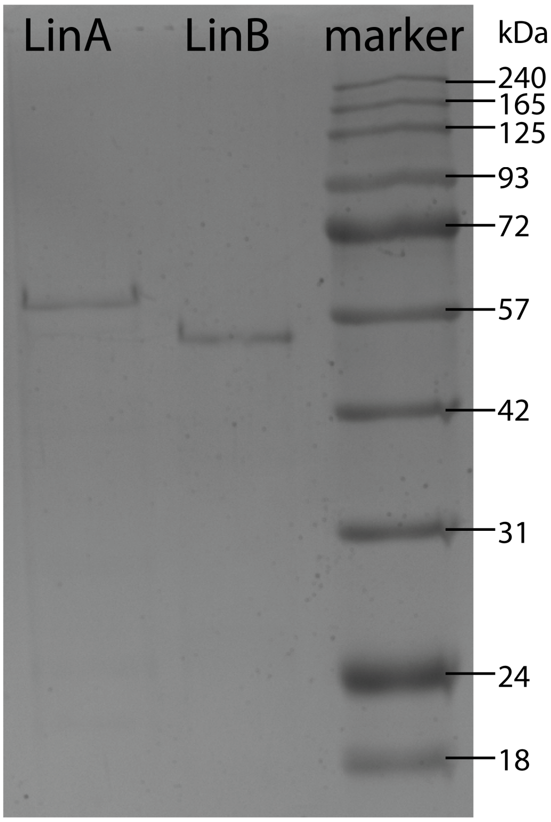

Figure S29. SDS polyacrylamid gel electrophoresis (SDS-PAGE) of purified His_6-tagged LinA and LinB. The calculated protein masses for LinA and LinB are 60.3 kDa (*C*-His_6-tagged: 61.8 kDa) and 53.5 kDa (*N*-His_6-tagged: 56.0 kDa), respectively.
