# Supplementary material for: Non-canonical two-step biosynthesis of anti-oomycete indole alkaloids in Kickxellales
Source: Fungal Biol Biotechnol. 2023 Sep 5;10:19. doi: 10.1186/s40694-023-00166-x (PMC10478498; doi:10.1186/s40694-023-00166-x)
Supplement: Supplementary file 35 — Additional file 35: Figure S31. Activity of LinB during competitive substrate supply. [file 40694_2023_166_MOESM35_ESM.pdf]

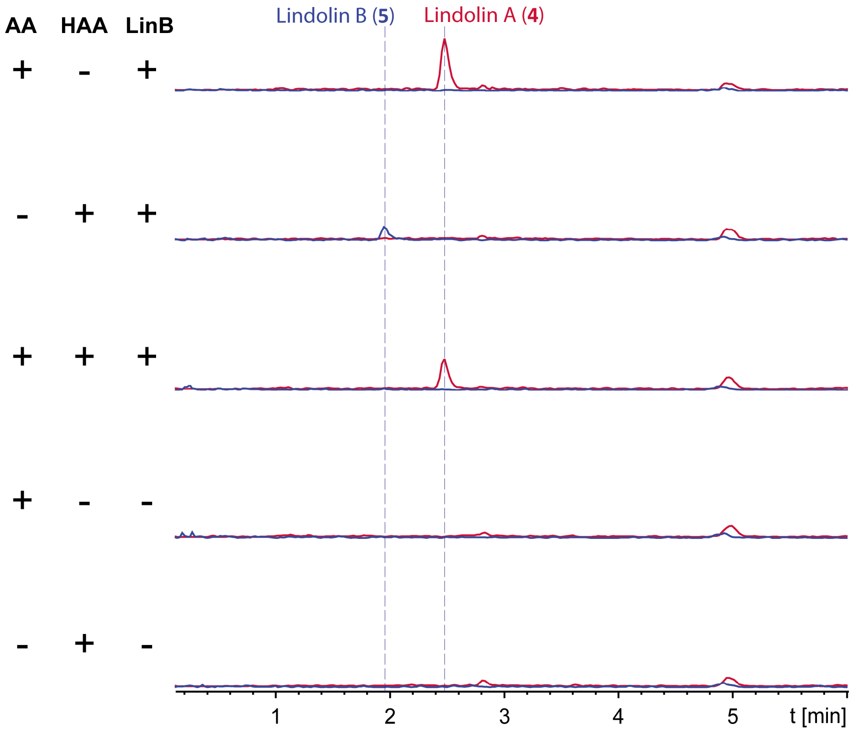

Figure S31. Activity of LinB during competitive substrate supply. LinB was incubated with anthranilic acid (AA) alone, 5-hydroxyanthranilic acid (HAA) alone or with both substrates equimolarly (1 mM each). Incubation without enzyme served as negative controls. **4** is the predominant product, when both substrates are provided. However, **5** is formed in absence of AA.
