# Supplementary material for: Non-canonical two-step biosynthesis of anti-oomycete indole alkaloids in Kickxellales
Source: Fungal Biol Biotechnol. 2023 Sep 5;10:19. doi: 10.1186/s40694-023-00166-x (PMC10478498; doi:10.1186/s40694-023-00166-x)
Supplement: Supplementary file 36 — Additional file 36: Figure S32. Determination of the optimal reaction conditions for LinB. [file 40694_2023_166_MOESM36_ESM.pdf]

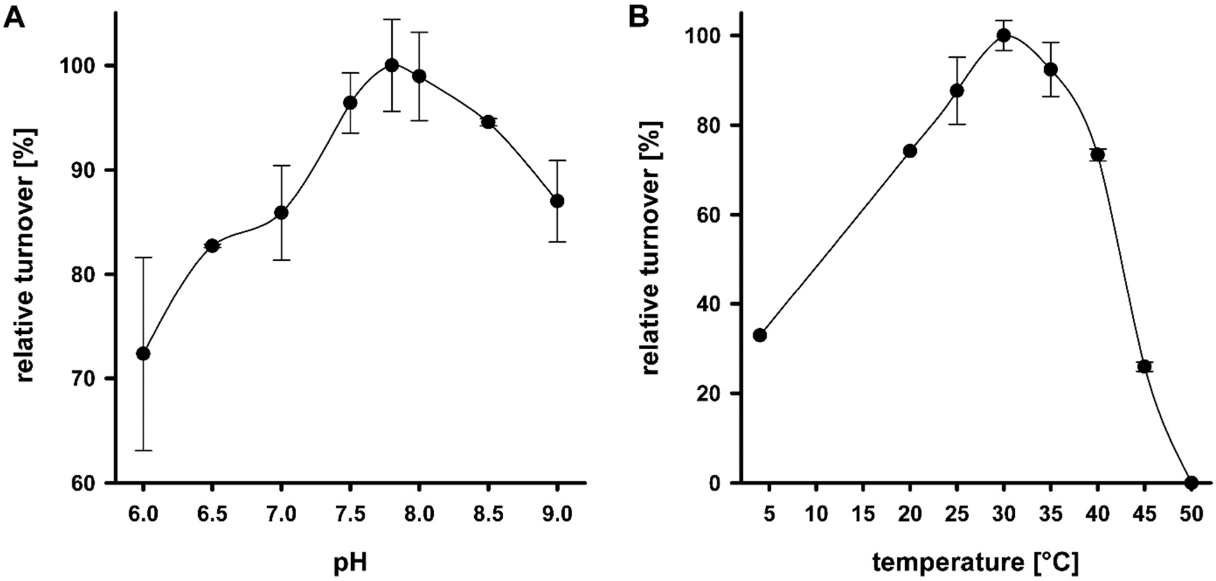

Figure S32. Determination of the optimal reaction conditions for LinB. The pH optimum (**A**) and the temperature optimum (**B**) were assigned at pH = 7.8 and ϑ = 30 °C, respectively.
