# Supplementary material for: Non-canonical two-step biosynthesis of anti-oomycete indole alkaloids in Kickxellales
Source: Fungal Biol Biotechnol. 2023 Sep 5;10:19. doi: 10.1186/s40694-023-00166-x (PMC10478498; doi:10.1186/s40694-023-00166-x)
Supplement: Supplementary file 38 — Additional file 38: Figure S34. Clinker comparison between linB gene loci in Kickxellales. [file 40694_2023_166_MOESM38_ESM.pdf]

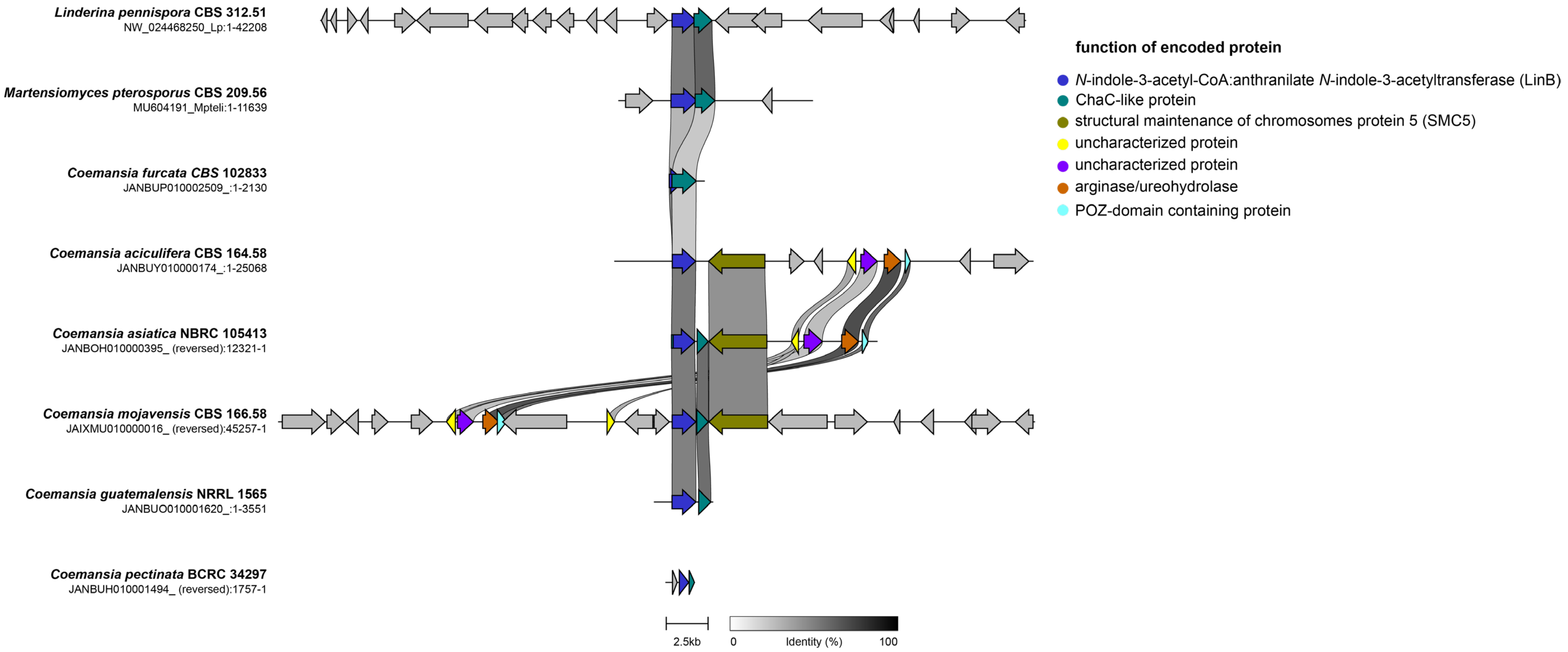

Figure S34. Clinker comparison between *linB* gene loci in Kickxellales. The conserved genomic neighborhood of *linB* ± 20 kbp is depicted, if available from the genome. Note, that some contigs might be truncated due to incomplete sequencing. Homologous genes are highlighted in identical colors while the links between those genes are presented according to the percentage identity. *linB* is highlighted in blue.
