# Supplementary material for: Non-canonical two-step biosynthesis of anti-oomycete indole alkaloids in Kickxellales
Source: Fungal Biol Biotechnol. 2023 Sep 5;10:19. doi: 10.1186/s40694-023-00166-x (PMC10478498; doi:10.1186/s40694-023-00166-x)
Supplement: Supplementary file 42 — Additional file 42: Figure S36. Percent identity depending on the alignment score of LinB-like proteins in SSN. [file 40694_2023_166_MOESM42_ESM.pdf]

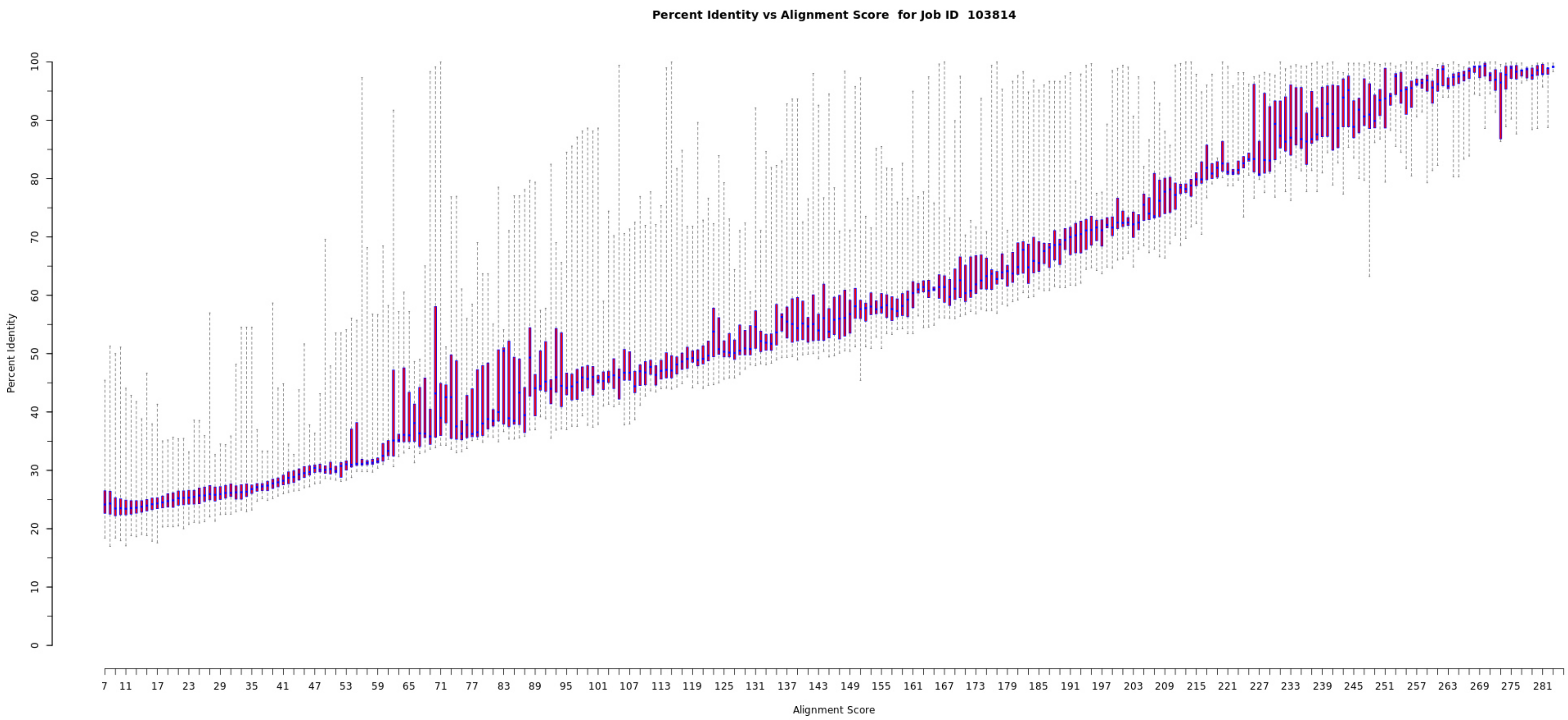

Figure S36. Percent identity depending on the alignment score of LinB-like proteins in SSN. Accession numbers of the used sequences are listed in Table S9.
