# Supplementary material for: Non-canonical two-step biosynthesis of anti-oomycete indole alkaloids in Kickxellales
Source: Fungal Biol Biotechnol. 2023 Sep 5;10:19. doi: 10.1186/s40694-023-00166-x (PMC10478498; doi:10.1186/s40694-023-00166-x)
Supplement: Supplementary file 43 — Additional file 43: Figure S37. Histogram of sequence lengths of LinB-like proteins used for SSN analysis. [file 40694_2023_166_MOESM43_ESM.pdf]

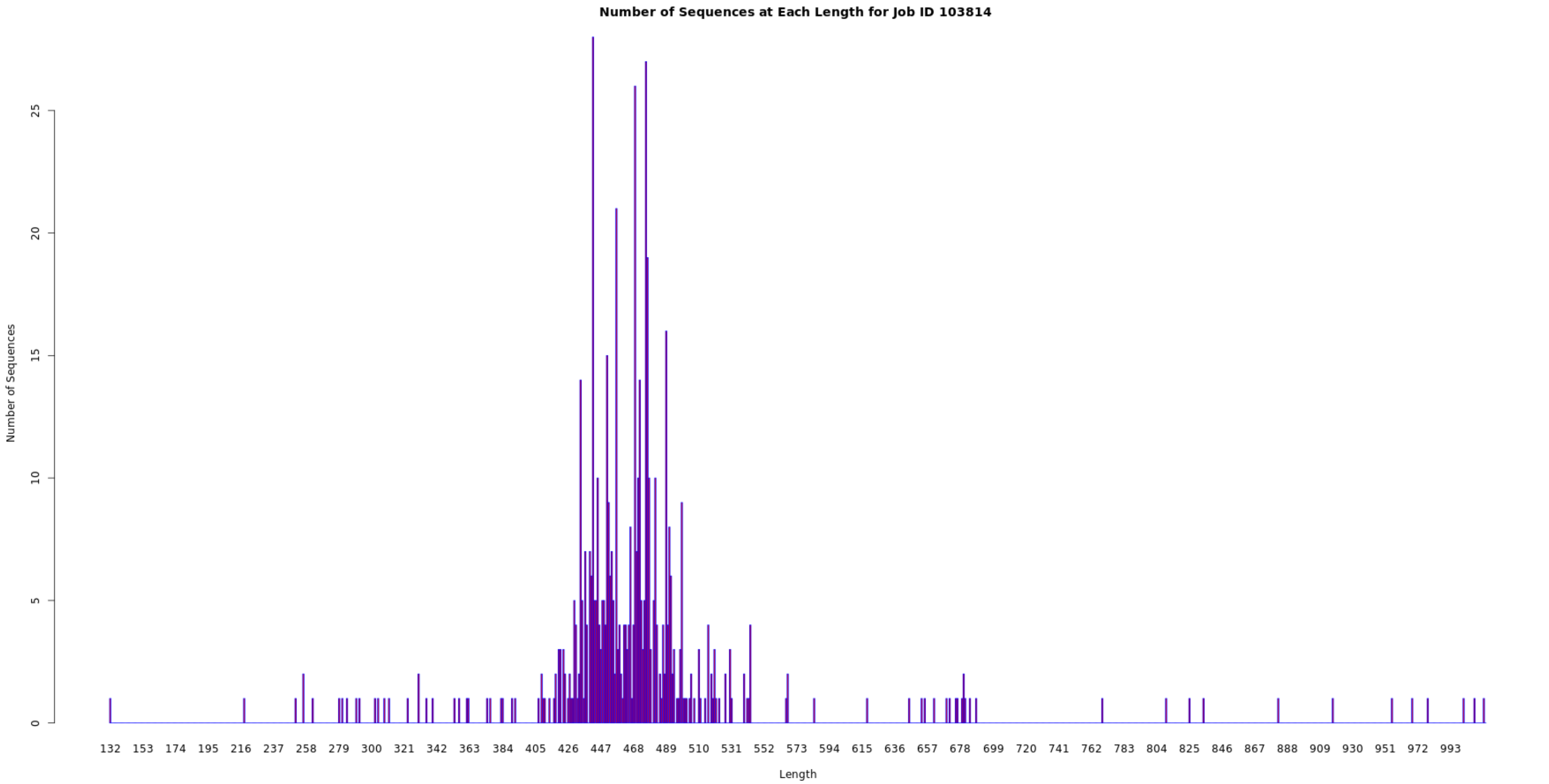

Figure S37. Histogram of sequence lengths of LinB-like proteins used for SSN analysis. Sequences with <400 and >600 amino acids were omitted from SNN analysis. Accession numbers of the used sequences are listed in Table S9.
