# Supplementary material for: Non-canonical two-step biosynthesis of anti-oomycete indole alkaloids in Kickxellales
Source: Fungal Biol Biotechnol. 2023 Sep 5;10:19. doi: 10.1186/s40694-023-00166-x (PMC10478498; doi:10.1186/s40694-023-00166-x)
Supplement: Supplementary file 44 — Additional file 44: Table S5. Chromatographic Methods. [file 40694_2023_166_MOESM44_ESM.pdf]

Table S5. Chromatographic Methods. ACN =acetonitrile, FA = formic acid.

	method 1	method 2	method 3	method 4	method 5	method 6	method 7
	GC/MS	UHPLC/MS	Flash chroma.	Flash chroma.	semipreparative HPLC	Flash chroma.	semipreparative HPLC
purpose	detection of **1, 2, 3**	detection of **3, 4, 5,** IAA-CoA	purification of **4** and **5**	purification of **4**	purification of **4**	purification of **5**	purification of **5**
system	Trace 1310 GC (Thermo)	Agilent 1290 Infinity II	C-810 (Büchi)	C-810 (Büchi)	Agilent 1200	C-810 (Büchi)	Agilent 1200
column	Trajan (SGE) BPX5 capillary column	Macherey&Nagel Nucleodur C18 Gravity	Büchi FP ID Si 40 g	Büchi FP ID C18 12g	Agilent Eclipse XDB-C18	Büchi FP ID C18 12g	Agilent Eclipse XDB-C18
column dimention	30 m, 0.25 mm inner diameter, 0.25 µm film	EC 50 x 2.0 mm	40 g 57 mL^{-1}<br>35-45 µm<br>53-80 Å	12 g 24 mL^{-1}<br>35-45 µm<br>53-80 Å	250 x 9.4 mm<br>5 µm	12 g 24 mL^{-1} 35-45 µm<br>53-80 Å	250 x 9.4 mm<br>5 µm
temp.	injector 200 °C<br>MS transfer 300 °C<br>ion source 250 °C	30 °C	20 °C	20 °C	12 °C	20 °C	12 °C
Eluent A	helium carrier gas	water + FA 0.1 %	hexane	water + FA 0.1 %	water + FA 0.1 %	water + FA 0.1 %	water + FA 0.1 %
Eluent B	-	ACN	ethylacetate	ACN	ACN	ACN	ACN
gradient (T or % B)	0–1 min: 40 °C<br>1–3 min: to 100 °C ($30\ °C \times min^{-1}$)<br>3–24 min: to 300 °C ($10\ °C \times min^{-1}$)	0-4 min: 5-72 %<br>4-4.5min: 72-95 %<br>4.5-5 min: 95 %	0-2 min: 0 %<br>2-24 min: 0-80 %<br>24-26 min: 80-100 %<br>26-32 min: 100 %	0-2 min: 0 %<br>2-4.9 min: 19 %<br>4.9-8 min: 35 %<br>8-10.7 min: 59 %<br>10.7-14.1 min: 79 %<br>14.1-19 min: 100 %	0-16 min: 45 %<br>16-20 min: 45-100 %<br>20-22.5 min: 100 %	0-1.8 min: 0 %<br>1.8-3.6 min: 10 %<br>3.6-5.4 min: 20 %<br>5.4-7.2 min: 30 %<br>7.2-9 min: 40 %<br>9-10.8 min: 50 %<br>10.8-12.6 min: 60 %<br>12.6-14.4 min: 80 %<br>14.4-18.4 min: 100 %	0-18 min: 35 %<br>18-18.5 min: 35-100 %<br>18.5-20 min: 100 %
flow	1.5 $mL \cdot min^{-1}$	1 mL min^{-1}	45 mL min^{-1}	30 mL min^{-1}	2.5 mL min^{-1}	30 mL min^{-1}	2 mL min^{-1}
detector	Thermo TSQ 9000 electron impact (EI)-triple quad mass spectrometer<br>(45 – 500 amu)	Agilent 6130 Single Quadrupol and<br>DAD<br>(λ = 254/280 nm)	DAD<br>(λ = 254 – 380 nm)	DAD<br>(λ = 254 – 380 nm)	DAD<br>(λ = 254 – 380 nm)	DAD<br>(λ = 254 – 380 nm)	DAD<br>(λ = 254 – 320 nm)
