# Supplementary material for: Non-canonical two-step biosynthesis of anti-oomycete indole alkaloids in Kickxellales
Source: Fungal Biol Biotechnol. 2023 Sep 5;10:19. doi: 10.1186/s40694-023-00166-x (PMC10478498; doi:10.1186/s40694-023-00166-x)
Supplement: Supplementary file 45 — Additional file 45: Table S6. Oligonucleotides used in this study. [file 40694_2023_166_MOESM45_ESM.pdf]

Table S6. Oligonucleotides used in this study.

oligonucleotide	5´-3´sequence	target gene	function	purpose	primer efficiency
oMG663<br>oMG664	TGAGAGAAATTCTTACGCTTCAGTTC<br>CATTAGCGCGCTCGATCTGC	*benA*	actin	qRT-PCR<br>(house-keeping gene)	0.95
oMG665<br>oMG666	TGGAGAAGTCTGCCTCGTAC<br>GTCGACAATGGACGAGTGG	*gpdA*	glycerinaldehyde-3-phosphat-dehydrogenase	qRT-PCR<br>(house-keeping gene)	1.02
oMG667<br>oMG668	AAGCCCATGTGCGTTGAGG<br>GGTGGTCTTGCCCGACTTC	*tefA*	transcription factor	qRT-PCR<br>(house-keeping gene)	1.03
oMG723<br>oMG724	CGTCATGAAGGACAGCGGC<br>GTGGTCGATAATCACAAACCCGC	*linA*	IAA—CoA ligase	qRT-PCR	1.00
oMG842<br>oMG843	GTTCAACCCACGCCCCAAC<br>CCAGCGTGACATACAAATGCAGC	*linB*	IAA-transferase	qRT-PCR	0.97
oMG749<br>oMG750	TATACCATGGTTCTCGTTTCTTTGCTCGAC<br>CCGCAAGCTTGGCCGCAACTGTTGCCTTG	*linA*	IAA—CoA ligase	production in *E. coli*<br>(*C*-terminal His_6-tag)	
oMG830<br>oMG831	TGCCGCGCGGCAGCCATATGGCTAGCATGGCCAGGCTCGACATGG<br>TGGTGCTCGAGTGCGGCCGCAAGCTTTCAGAATTCGCTGACAACAGAGC	*linB*	IAA-transferase	production in *E. coli*<br>(*N*-terminal His_6-tag)	
