# Supplementary material for: Non-canonical two-step biosynthesis of anti-oomycete indole alkaloids in Kickxellales
Source: Fungal Biol Biotechnol. 2023 Sep 5;10:19. doi: 10.1186/s40694-023-00166-x (PMC10478498; doi:10.1186/s40694-023-00166-x)
Supplement: Supplementary file 46 — Additional file 46: Table S7. Plasmids used in this study. [file 40694_2023_166_MOESM46_ESM.pdf]

Table S7. Plasmids used in this study.

expression plasmid	expressed gene	vector backbone	host	induction
pNH07	*Linderina pennispora linA* (cDNA)	pET28a (+)	*E. coli* SoluBL21	1 mM IPTG, 16 °C, 16 h
pNH08	*Linderina pennispora linB* (cDNA)	pET28a (+)	*E. coli* BL21	1 mM IPTG, 16 °C, 16 h
